# Design, Fabrication and Characterization of Multi‐Yolk@Shell NiCuFe_2_O_4_@mSiO_2_ Mesoporous Nanocomposite Spheres for the Synthesis of Pyrimido‐Quinolines under Solvent‐Free Conditions

**DOI:** 10.1002/open.202300053

**Published:** 2023-09-08

**Authors:** Somayeh Kazempour, Hossein Naeimi

**Affiliations:** ^1^ Department of Organic Chemistry University of Kashan 87317-51167 Kashan Iran

**Keywords:** mesoporous, multi-shell nanoparticles, pyrimidoquinoline, recyclability, spherical morphology

## Abstract

Multi‐yolk@shell mesoporous silica spheres are becoming more and more attractive as high‐performance catalysts because of their high surface areas, variable pore sizes, and low densities. In this work, a NiCuFe_2_O_4_ magnetic core with a shell of mesoporous silica mesoporous has been prepared in an easy two‐step procedure. The prepared multi‐yolk@shell NiCuFe_2_O_4_@mSiO_2_ spheres were characterized by using FT‐IR, XRD, VSM, EDX, BET, FE‐SEM and HR‐TEM techniques. These unique multi‐yolk@shell NiCuFe_2_O_4_@mSiO_2_ spheres demonstrated high catalytic activity for the synthesis of pyrimidoquinolines. Also, this method exposes obvious benefits such as catalyst recyclability, easy reaction condition, simplicity of work up, high product yields and short reaction times.

## Graphical abstract

A NiCuFe_2_O_4_ magnetic core with mesoporous silica shell has been prepared in two easy steps. This unique multi‐yolk@shell NiCuFe_2_O_4_ @mSiO_2_ composite demonstrated high catalytic activity for the synthesis of pyrimidoquinolines.

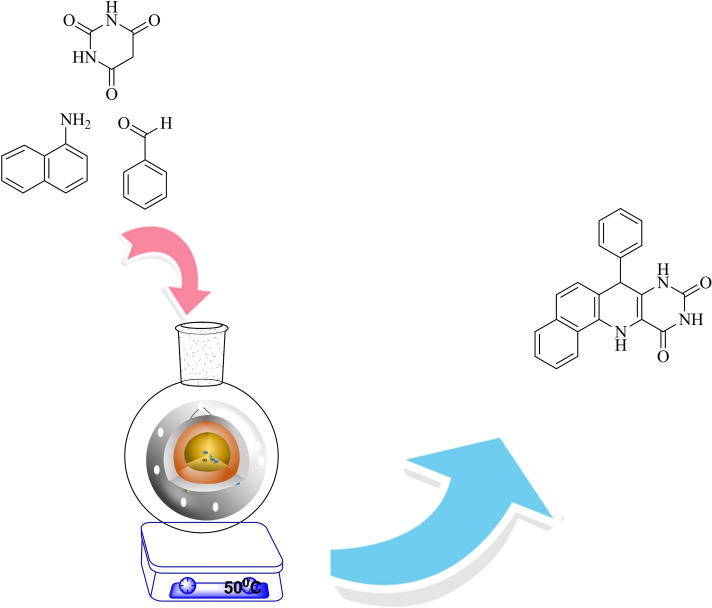

## Introduction

Hollow inorganic nanoparticles that contain a nanostructure void space enclosed in a porous inorganic nanoshell have been of considerable interest, both from a research and an industrial viewpoint.[^1^, ^2^] Their unique features, which result from the presence of interior void space, have been evidenced through research in recent years. These nanocomposites are effective in several applications such as; nano structures,[^3^, ^4^] contrast agents for molecular imaging,[^5^, ^6^] drug‐delivery equipment,^[7]^ efficient energy and gas‐storage materials.^[8]^ Contrarily, their vast surface areas make it possible for them to carry a lot of useful species, including catalysts and molecular imaging agents, which enable reactive catalysis and sensitive diagnostics. It is possible to load, store, and distribute functional molecules such as drug molecules, to the target region using the void area protective shell. Additionally, by using their permeable porous nanoshell, we can carefully control the condition and making the product. The nanoparticles (NPs) are considered as the structure blocks for different applications of nanotechnology.The researchers have been examined the benefits of structurally distinct NPs over basic spherical particles. Because of their increased surface area, improved functionaliz‐ability, and other physicochemical features, the advancement of NPs research is advancing in the directions of anisotropic form,^[9]^ hollow,^[10]^ core/shell,^[11]^ and yolk/shell^[12]^ structures.

Yolk‐shell materials are group of hollow structures, which features an inside flexible core and an outside hollow shell, that has attracted much attention.^[13]^ The yolk‐shell structures can create a catalysis field, with three crucial features that should be emphasized: (1) the complete place in the active core in the shell, creates somewhat balance between efficiency and stability; (2) the presence of void greatly increases the space for the occurrence of catalytic reaction and mass transfer; (3) The capacity to manipulate the shell, yolk, void, or a mixture of these, allows for the dynamic and flexible adjustment of the catalytic performance, stability, reusability, and even synergistic effect generated multifunction.

Magnetic core with mesoporous silica shell has lately emerged as a family of novel functional nanomaterials and has received much attention because it has a combination of magnetic nanomaterials properties and the outstanding properties of mesoporous materials such as; high surface area, uniform structure, and highly ordered mesoporous.^[14]^

Magnetic core attracted researchers because of easily separated in the chemical reactions. These used as a metallic and multimetallic magnetic catalyst in the chemical reactions such as; NiFe_2_O_4_,^[15]^ CuFe_2_O_4_,^[16]^ NiCuFe_2_O_4_,^[17]^ Fe_3_O_4_ ^[18]^ and CoFe_2_O_4_.^[19]^ The magnetic mesoporous silica shell can be created through either soft templating method based on surfactants.^[20]^ The role of the mesoporous shell in hollow structures is to prevent the accumulation of nanoparticles, creating a highly available surface area.[^21^, ^22^, ^23^]

Since the products are synthesized in a single step and diversity may be obtained by simply changing each component, multicomponent reactions (MCRs) are an effective technique for the synthesis of organic molecules.^[24]^ MCRs have received a lot of attention because of how simple they are to use and how well they perform.[^25^, ^26^] A group of multicomponent reactions is called the synthetic method of quinolines.

**Figure 1:**
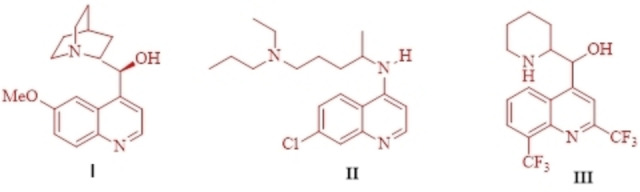
The structures of quinine (I), chloroquine (II), and mefloquine (III).

Some of the quinoline ring systems, which contain medications including quinine (I), chloroquine (II), and mefloquine (III) is used as an effective malaria treatment^[27]^ (Figure 1). Furthermore, some quinoline‐based substances exhibit potent anticancer action.^[28]^

The adaptability of quinoline in synthetic chemistry, which allows for the creation of a large number of structurally varied derivatives, has further aided this wide range of biological and biochemical functions. Since quinolines were synthesized last year, they have issues with low yield, slow reactions, and long reaction times. In this protocol, we hope to report the design and preparation of a novel reactive heterogeneous catalyst for the synthesis of pyrimidoquinoline derivatives under solvent‐free conditions.

## Results and Discussion

### Preparation and chracterization of catalyst

**Scheme 1:**
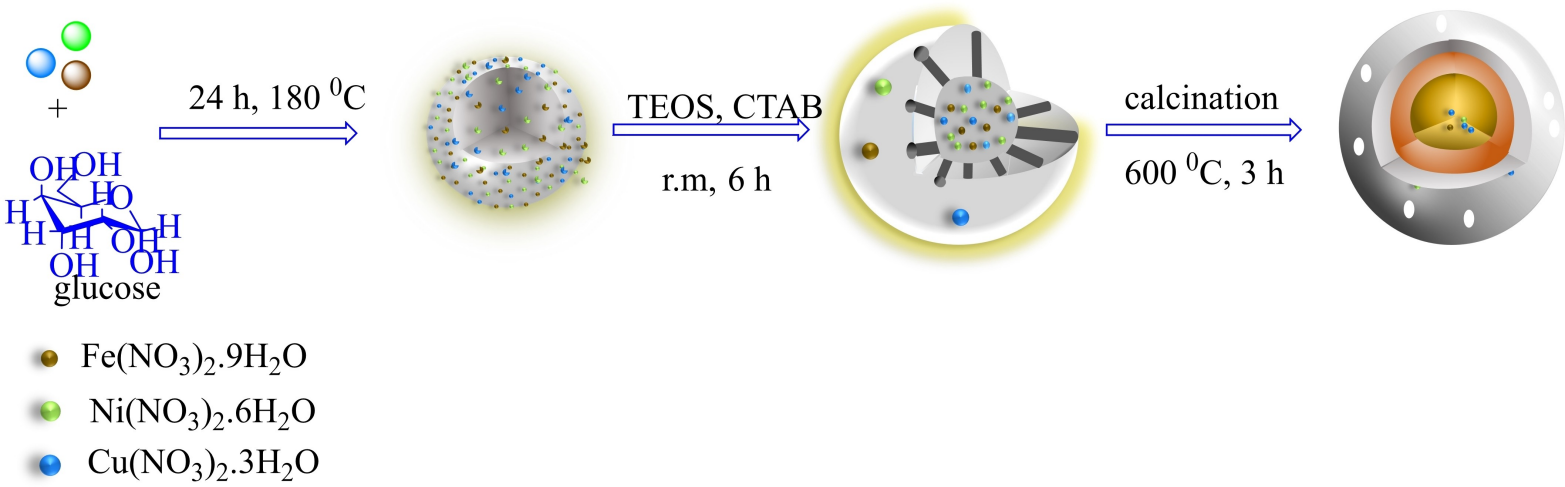
Preparation of multi yolk@shell NiCuFe_2_O_4_@mSiO_2_ spheres.

The multi‐yolk@shell NiCuFe_2_O_4_@mSiO_2_ composite has been designed and easily made by a procedure in three steps. In the first step, Ni(NO_3_)_2_.6H_2_O, Cu(NO_3_)_2_ ⋅ 3H_2_O, and Fe(NO_3_)_2_ ⋅ 9H_2_O were mixed in a solution of glucose at room temperature for 30 minutes by magnetic stirring. Then, the reaction mixture was added to autoclave and heated in an oven at 180 °C for 24 h during the hydrothermal formation of carbon spheres. In the second step, the obtained black solid in the first step, CTAB and NH_3_ ⋅ H_2_O was dispersed into H_2_O and ethanol as solvent. Then, magnetic stirring was applied for 30 min and the TEOS was added into the system. The mixture was maintained at room temperature for 6.0 h. Then, the product was collected by using an external magnet and dried in an oven for 6.0 h. In the third step, NiCuFe_2_O_4_@C@SiO_2_ solid was recovered, calcinated at 600 °C for 3.0 h, and the multi yolk@shell NiCuFe_2_O_4_ @ mSiO_2_ was obtained (Scheme 1).

**Figure 2:**
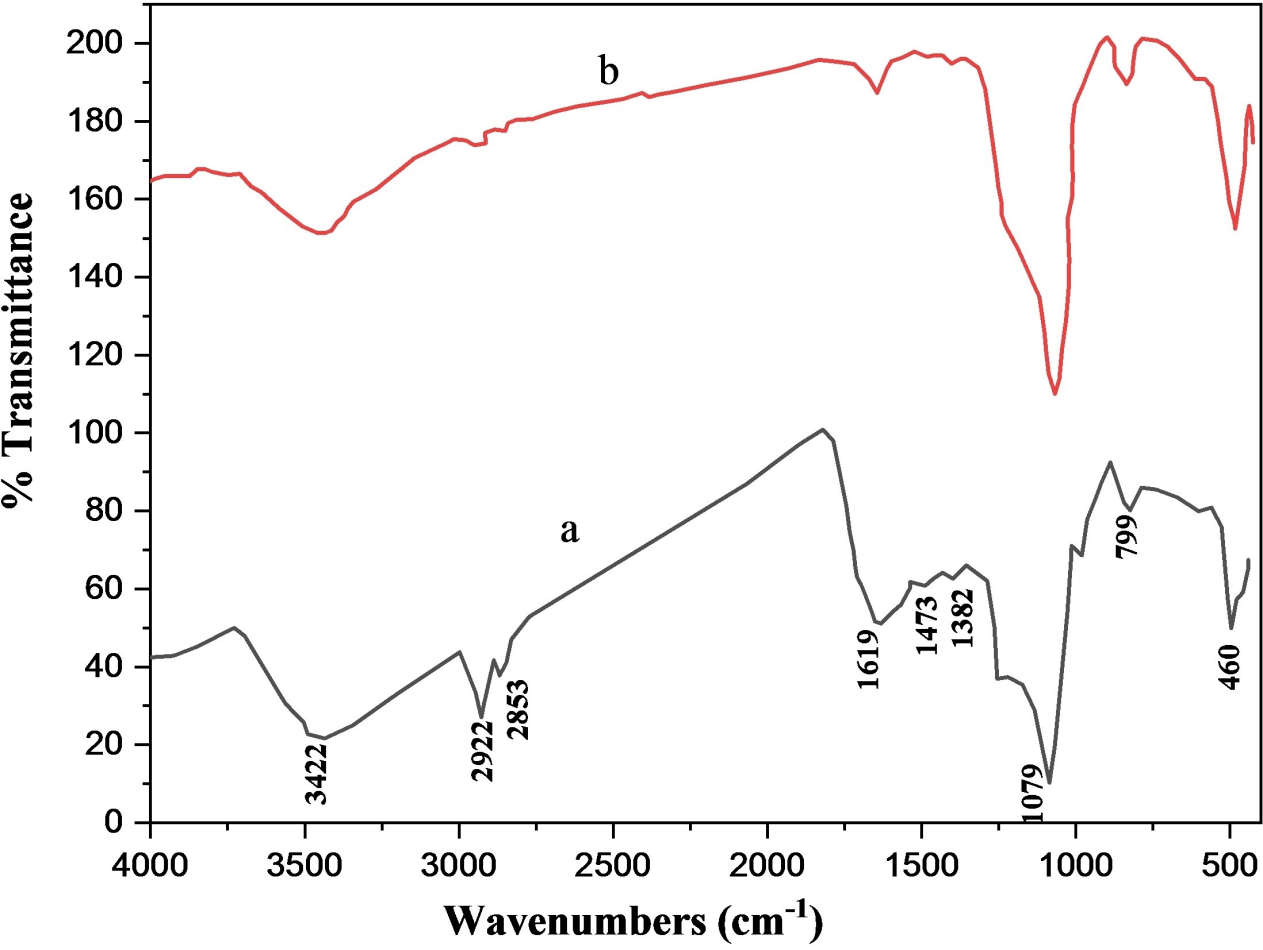
FT‐IR spectra of the multi yolk@shell NiCuFe_2_O_4_ spheres, a) before and b) after calcination.

The prepared catalyst was characterized by FT‐IR, XRD, FE‐SEM, EDX, HR‐TEM, VSM and BET techniques. Figure 2 shows the FT‐IR spectra of hollow catalyst before and after calcination. Figure 2(a) shows the NiCuFe_2_O_4_@C@SiO_2_ catalyst that was appropriated before calcination. The peaks of 3422 and 1619 cm^−1^ show the stretching vibration of the O−H bond and bending vibration of H−O−H bond. The peaks of the 2922 and 2853 cm^−1^ can be assigned to the stretching vibration of the C−H bond, and the peaks of 1473, 1382 cm^−1^ identify the bending vibration of the C−H bond and the stretching vibration of the C−O bond which show the template carbon is formed and CTAB is in the NiCuFe_2_O_4_@C@SiO_2_ catalyst. The peaks that appeared at 460 and 794 cm^−1^ can be related to the Ni−O, Cu−O, and Fe−O bonds. The stretching vibration of the Si−O−Si bond appeared in 1079 cm^−1^.^[29]^ Figure 1(b) shows the FT‐IR spectra of NiCuFe_2_O_4_@C@SiO_2_ catalyst after calcination. In comparison, the absence of the peaks in 2922, 2853, 1473 and 1382 cm^−1^ indicated that the calcination is completely done and the NiCuFe_2_O_4_ @ mSiO_2_ was formed.

**Figure 3:**
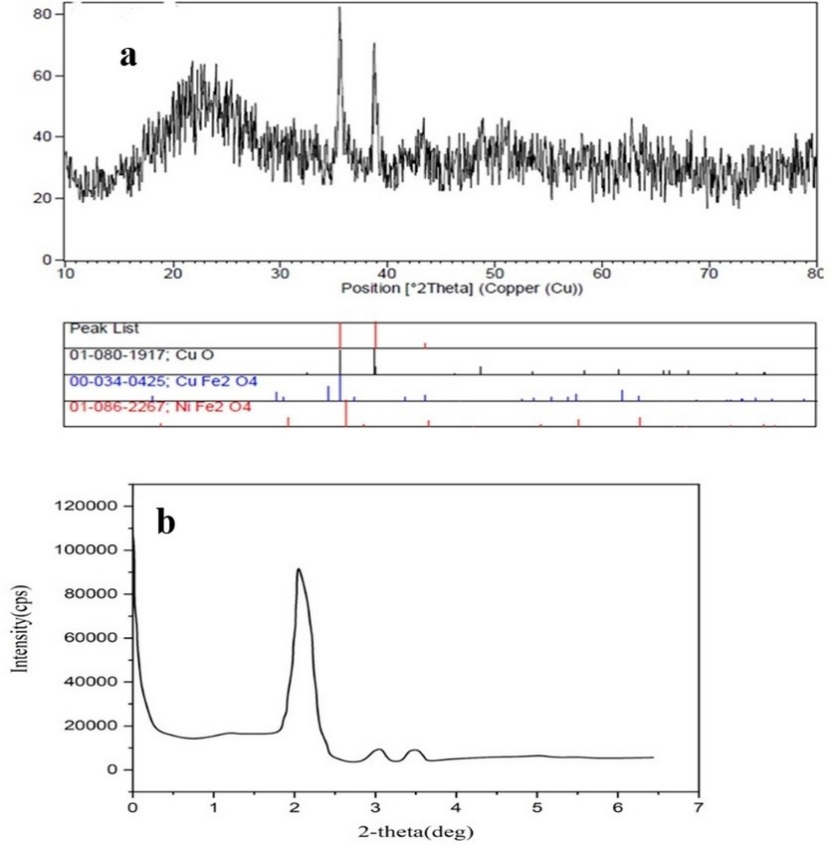
(a) XRD pattern of the NiCuFe_2_O_4_@mSiO_2_spheres and (b) low‐angle XRD of the NiCuFe_2_O_4_@mSiO_2_ spheres.

The XRD pattern of multi yolk@shell NiCuFe_2_O_4_ is shown in Figure 3 in which the related peaks are visible. The presence of the peaks at 2Θ=20.2°, 33.0°, and 36.6° proves that the catalyst is made regularly. The XRD pattern of this multi yolk@shell catalyst (Figure 3a) was compared with that of CuFe_2_O_4_ (JCPDS No. 77‐0010) and NiFe_2_O_4_ (JCPDS file No. 10‐325). It is evident that the reflections of CuNiFe_2_O_4_ are in excellent accordance with the JCPDS cards of CuFe_2_O_4_ and NiFe_2_O_4_ nanoparticles. However, it is also apparent that peaks from the impurity phases can be detected in this XRD pattern. Figure 3b shows the low‐angle XRD of catalyst and the presence of the sharp peak at 2Θ indicated that the multi yolk@shell NiCuFe_2_O_4_ mesopuoros silica catalyst is made.

**Figure 4:**
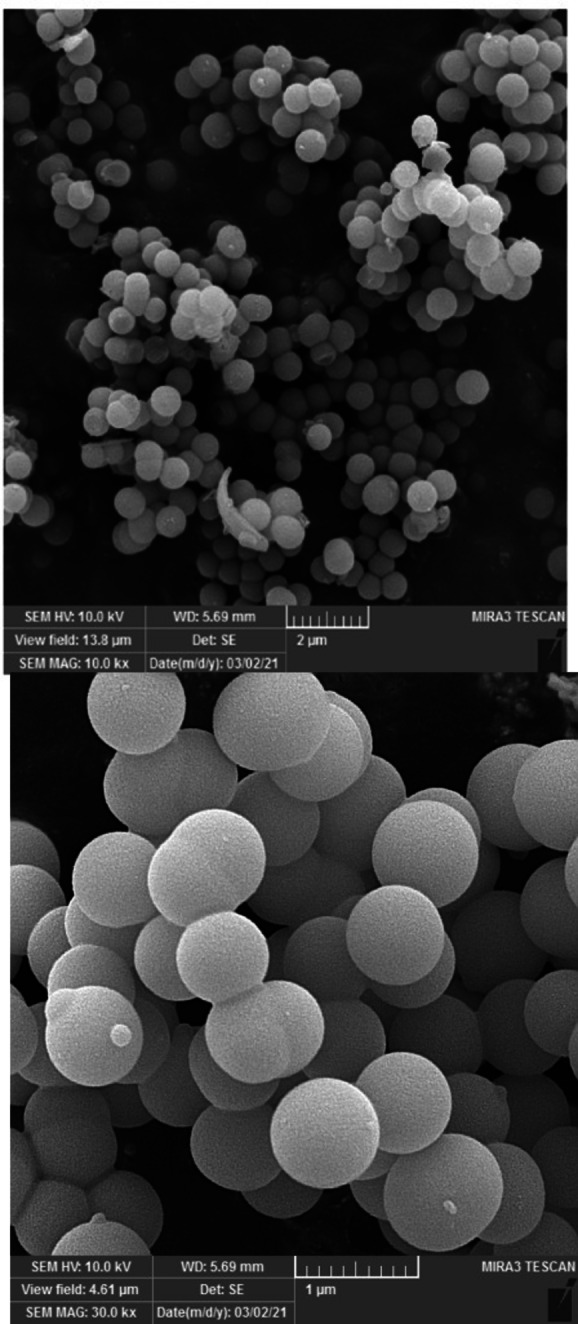
FE‐SEM images of the multi yolk@shell NiCuFe_2_O_4_@mSiO_2_ nanospheres.

Figure 4 shows field emission scanning electron microscopy (FE‐SEM) images for hollow multishell NiCuFe_2_O_4_@mSiO_2_ spheres catalyst. The FE‐SEM images (Figure 4) indicated that the catalyst is made up of uniform, spheres, and hollow.

**Figure 5:**
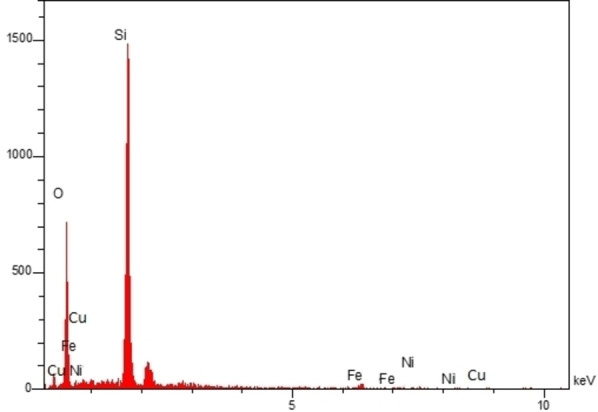
EDX spectrum of the multi yolk@shellNiCuFe_2_O_4_@mSiO_2_ spheres.

Also, Figure 5 demonstrates the EDX analysis of the hollow multi‐shell NiCuFe_2_O_4_@mSiO_2_ spheres. This template indicated that the nanospheres included the Ni, Cu, O, Si, and Fe elements with loading of 1.00, 1.11, 61.15, 34.65 and 2.09 %, respectively. This finding is in accordance with the FT‐IR results.

Moreover, to determine the amount of Fe, Ni and Cu in the catalyst structure, inductively coupled plasma atomic emission spectroscopy (ICP‐OES) was used. The results showed 1.21 ⋅ 10^−4^ mol g^−1^ of Fe, 0.28 ⋅ 10^−4^ mol g^−1^ of Ni and 0.39 ⋅ 10^−4^ mol g^−1^ of Cu were loaded on the hollow multishell NiCuFe_2_O_4_@mSiO_2_ spheres as a nanocatalyst.

**Figure 6:**
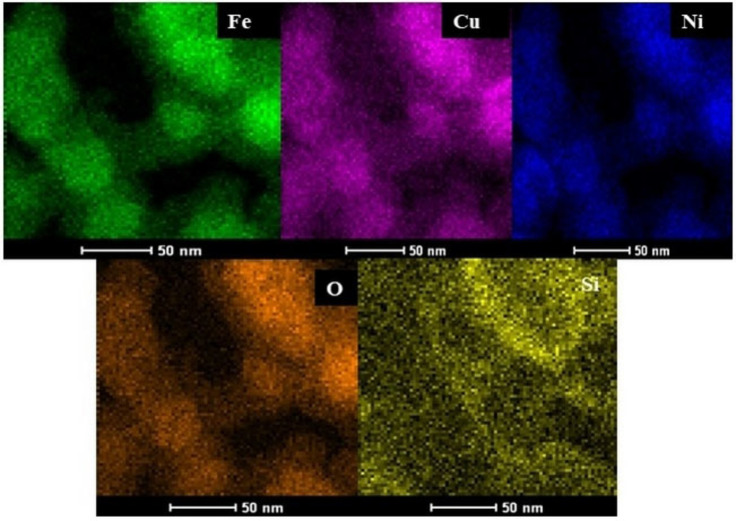
Elemental mapping analysis of yolk@shell NiCuFe_2_O_4_@mSiO_2_ catalyst.

Furthermore, the results of elemental mapping analysis indicate the Fe, Cu, Ni, O, and Si elements (Figure 6). This result is in agreement with the EDX analysis.

**Figure 7:**
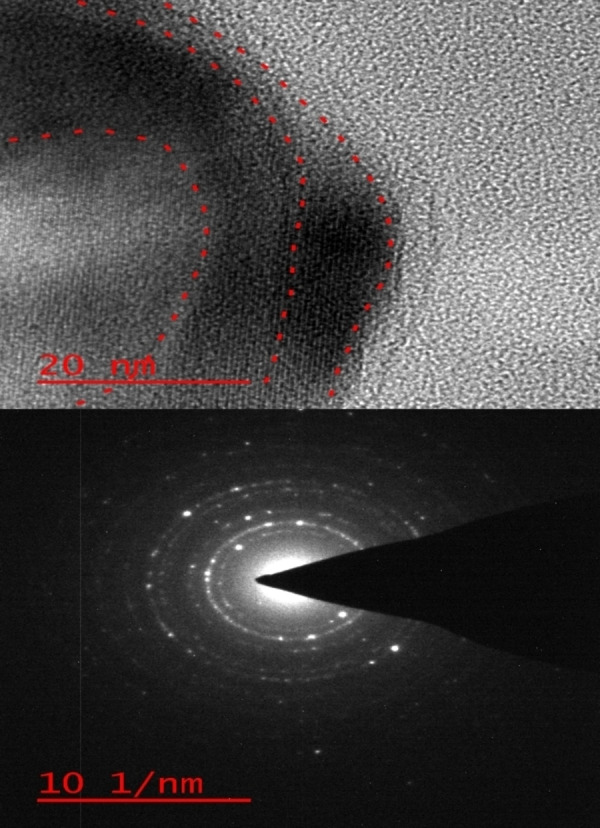
The HR‐TEM images of the multi yolk@shellNiCuFe_2_O_4_@mSiO_2_.

The high resolution TEM (HR‐TEM) images of the multi yolk@shellNiCuFe_2_O_4_@mSiO_2_ spheres indicates the structure of the catalyst in Figure 7. These images show that the catalyst was made in a sphere uniform, multishell, yolk‐shell and multimetal because of image shows the different sizes and avoide space around particles.

**Figure 8:**
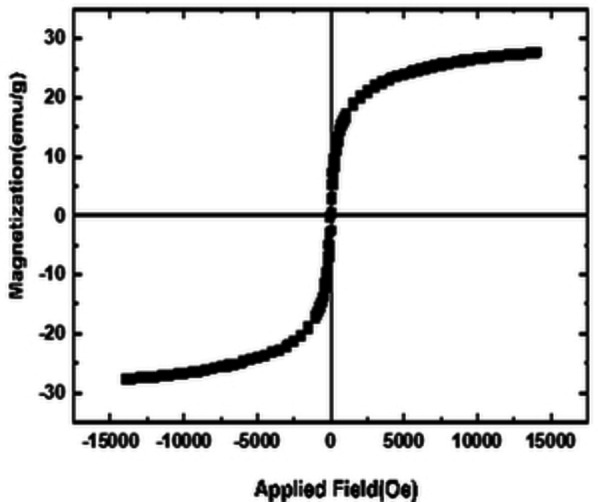
The VSM analysis of the multi yolk@shellNiCuFe_2_O_4_@mSiO_2_ spheres.

A vibrating sample magnetometer (VSM) has been used to explore the magnetic properties of various materials NiCuFe_2_O_4_@mSiO_2_ spheres. The magnetization pattern are shown in Figure 8. The multi yolk@shellNiCuFe_2_O_4_@mSiO_2_ spheres have a 28.1 emu per g magnetic valence.

**Figure 9:**
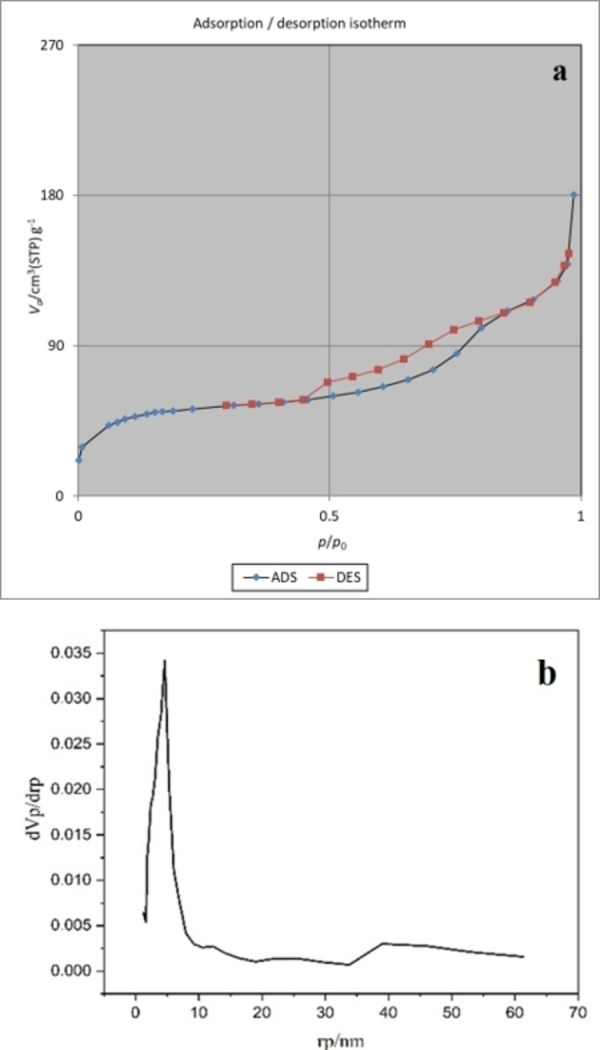
The nitrogen adsorption–desorption isotherm (a) and corresponding BJH pore size (b) of multiyolk@shellNiCuF_2_O_4_@mSiO_2_.

The nitrogen adsorption–desorption isotherm of the hollow multishell NiCuFe_2_O_4_@mSiO_2_ and corresponding BJH pore size distribution is shown in Figure 9. BJH analysis indicated an average pore size of 4.6 nm which indicated the catalyst is mesoporous (Figure 9b). Due to mesoporous silica, the available area surface at 180.51 m^2^/g is higher than for the catalyst reported without mesoporous silica (Figure 9a).^[30]^

### Investigation of catalytic activity

In order to assess its ability as a catalyst for the production of pyrimidoquinoline, multi yolk@shellNiCuF_2_O_4_@mSiO_2_ was investigated (Table 1) in the reaction of barbituric acid, 1‐naphthylamine and the 4‐Br‐benzaldehyde as a reaction model. Thus, an investigation of the influence of heat, reactivity of solvent, and nanocatalyst amounts on the reaction speed was conducted (Table 1). The results shows low product yields when using toluene, CH_3_CN and, DMSO as solvents (Table 1, entries 3–5). In this research, the best conditions for the model reaction at 50 °C to create pyrimidoquinolines were reported to be solvent‐free (Table 1, entry 7).

**Table 1:** Optimization of the reaction conditions.^[a]^

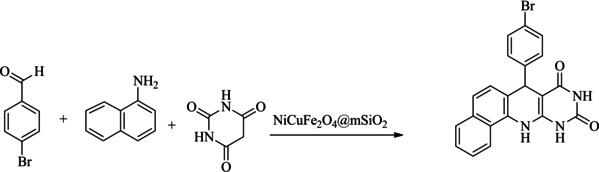					
Entry	Solvent	T (°C)	Catalyst (mg)	Time (min)	Yield (%)^[b]^
1	EtOH	Reflux	2	250	92
2	MeOH	Reflux	2	260	94
3	CH_3_CN	Reflux	2	290	–
4	DMSO	140	2	150	–
5	Toluene	Reflux	2	420	–
6	H_2_O/EtOH	Reflux	4	260	90
7	Solvent‐free	50	1	20	98

[a] Reaction conditions: 4‐Br‐benzaldehyde (1 mmol), 1‐naphthylamine (1 mmol), barbituric acid (1 mmol). [b] Isolated yield.

Several kinds of catalysts for this reaction were investigated once the reaction parameters were optimized (Table 2). When the present catalyst was compared with the other catalysts, such as; NiFe_2_O_4_, CuFe_2_O_4_, CoFe_2_O_4_ and Fe_3_O_4_ nanoparticles, it was found that the reaction in the presence of multi yolk@shell NiCuFe_2_O_4_ @mSiO_2_ spheres catalyst, was obtained the highest product yield. As a result, multi yolk@shell NiCuFe_2_O_4_@mSiO_2_ spheres are preferable to other catalysts because of short reaction time, low catalyst loading and highest product yield in the reaction.

**Table 2:** Catalytic activity of different catalysts for synthesis of primidoquinoline.

Entry	Catalyst	Time (min)	Yield (%)
1	NiFe_2_O_4_	210	88
2	CuFe_2_O_4_	210	86
3	Fe_3_O_4_	180	87
4	CoFe_2_O_4_	210	86
5	multi yolk@shell NiCuFe_2_O_4_@mSiO_2_	20	98

Following the adjustment of the reaction conditions including temperature, catalyst loading, and different solvents, the development and aim of our methodology for the production of various pyrimidoquinolines were carried out. This study looked at how different benzaldehydes reacted with 1‐naphthylamine, barbituric acid, and the corresponding results are summarized in Table 3. It can be seen from this table that 1‐naphthylamine and barbituric acid effectively reacted with a variety of aldehydes, including those having electron‐withdrawing and electron‐donating substituents. The results showed that the appropriate pyrimidoquinolines, including different substituents, are synthesized in the majority of cases with high yields and short reaction times. The reaction was absolutely uniform.

**Table 3:** Synthesis of primidoquinoline derivatives.^[a]^

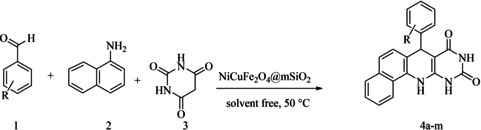					
Entry	Aldehyde	Product	Time (min)	Yield^[b]^ (%)	m. p. (°C)
1	H	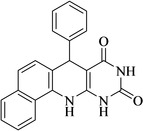	30	96	>300
2	2‐Cl	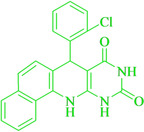	30	95	>300
3	4‐CH_3_	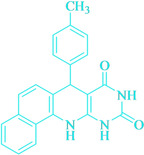	30	95	>300
4	2‐F	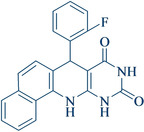	25	97	>300
5	4‐F	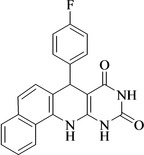	20	96	>300
6	4‐OH	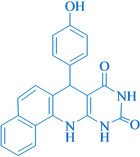	25	96	>300
7	3‐Br	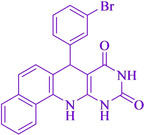	25	97	>300
8	4‐Cl	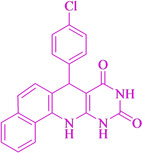	20	98	>300
9	3‐NO_2_	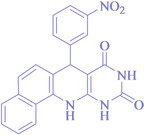	25	97	>300
10	4‐NO_2_	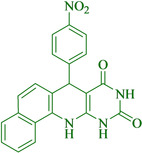	20	98	>300
11	4‐Br	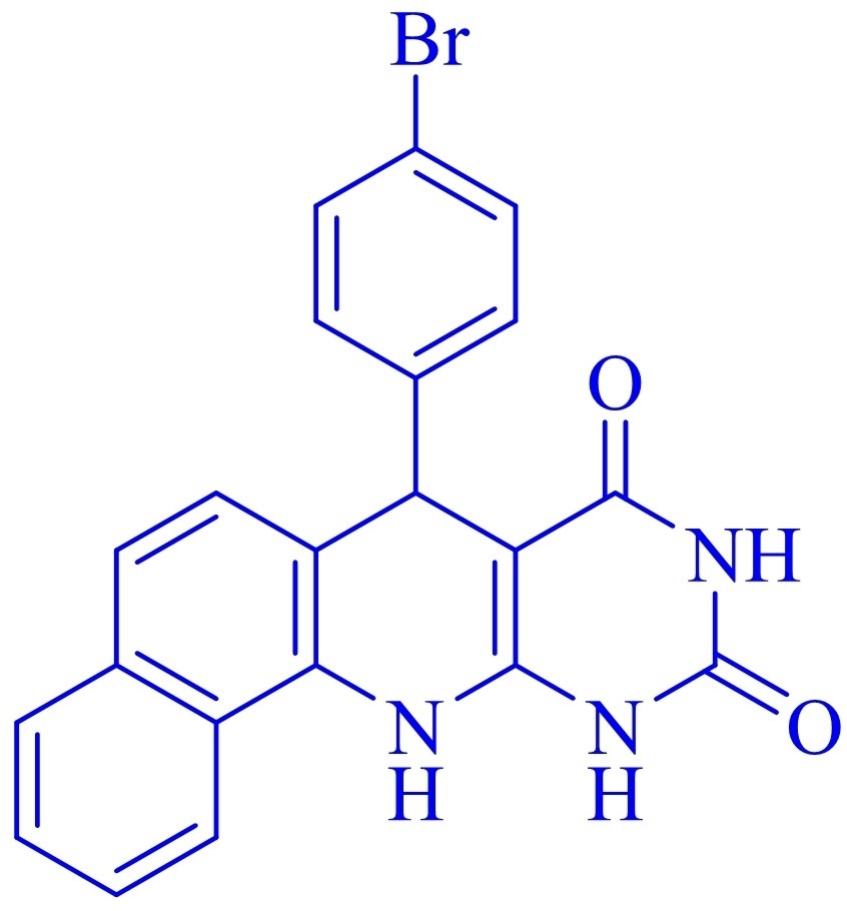	20	98	>300
12	2‐OH,5‐Br	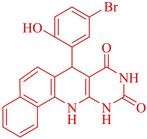	25	96	>300
13	2,3‐di‐OH	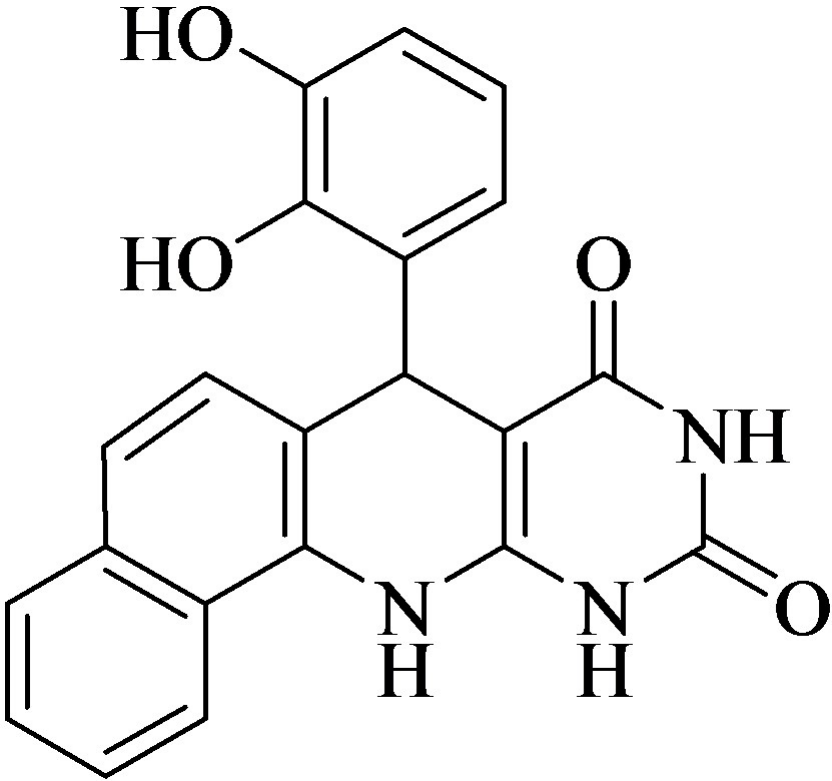	25	96	>300

[a] Reaction conditions: benzaldehyde (1 mmol), barbituric acid (1 mmol), 1‐naphthylamine (1 mmol) and multi yolk@shellNiCuFe_2_O_4_@mSiO_2_ (1 mg). [b] Isolatedyield.

### Proposed reaction mechanism

**Scheme 2:**
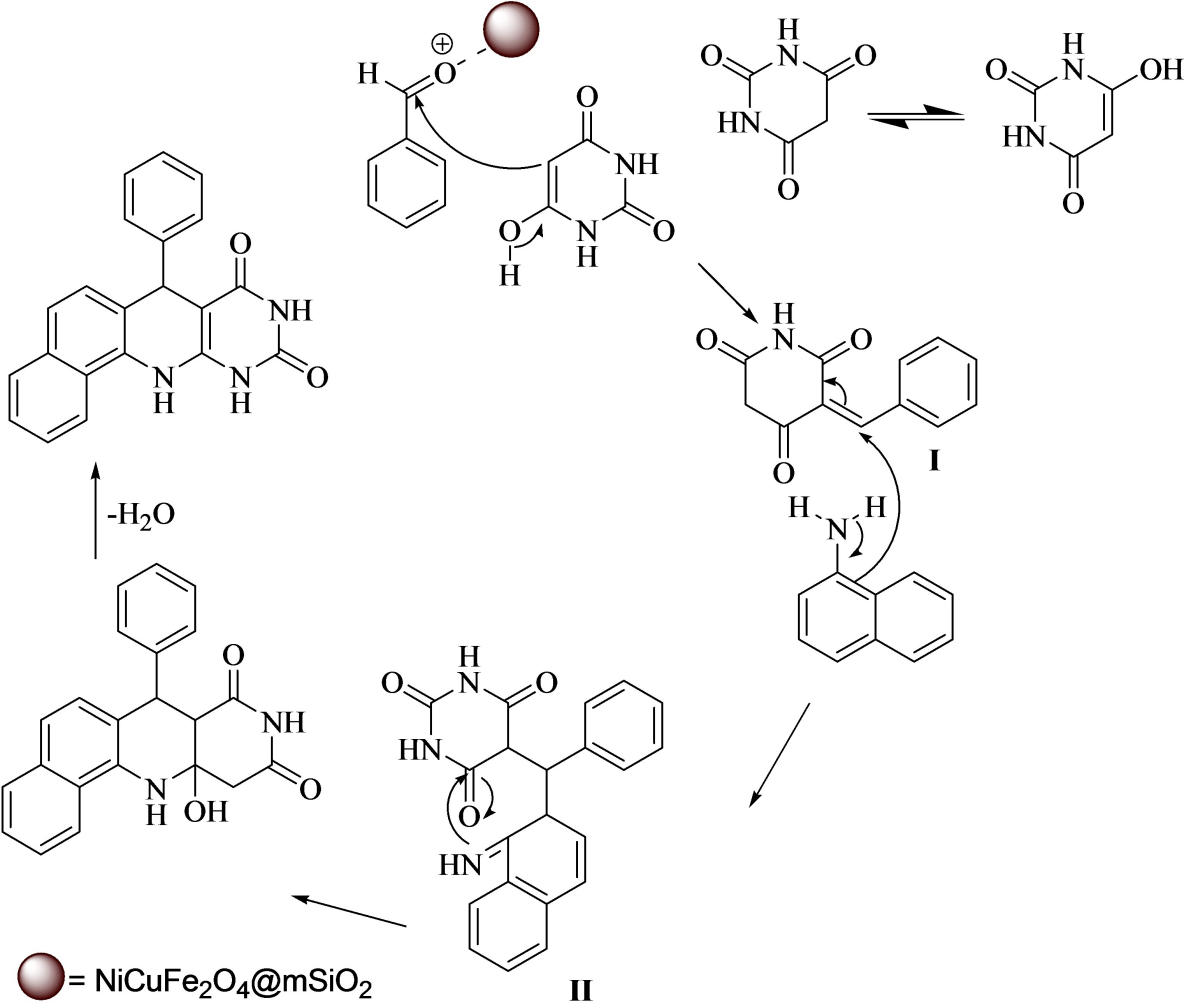
The proposed reaction mechanism for the formation of pyrimidoquinolines.

Scheme 2 suggests a plausible mechanism for the formation of pyrimidoquinoline. In this reaction, at the first the benzaldehyde is activated by the catalyst, then the activated benzaldehyde reacts with barbituric acid and an intermediate I created. Then, 1‐naphthylamine reacts with the I, and an intermediate II is formed. Finally, the intermediate II through cyclization and dehydration produced the target product.

### Reusability of the catalyst

**Figure 10:**
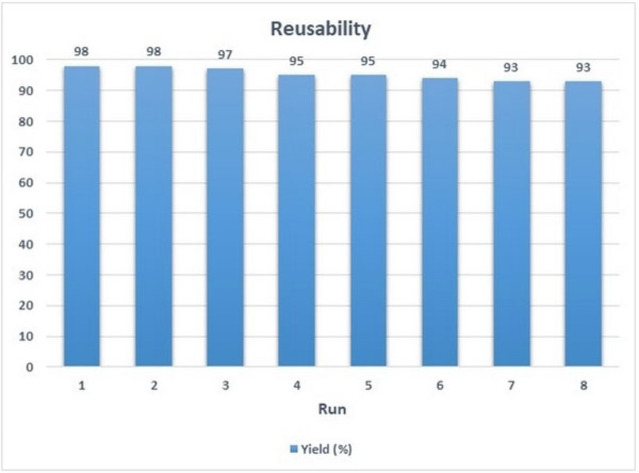
Reusability of the multi yolk@shell NiCuFe_2_O_4_@mSiO_2_ spheres over 8 repetitions of the reaction.

The catalyst after completion of the reaction separated by an external magnet, washed with ethanol and dried. The benzaldehyde with 1‐naphthyl amine and barbituric acid was chosen as a model reaction to measure the catalyst reusability. Figure 10 shows the results for using the recovered hollow triple‐shell NiCuFe_2_O_4_@mSiO_2_ nanospheres in the reaction after 8 times without loss of activity.

## Conclusions

In this research, a multi yolk@shell NiCuFe_2_O_4_@mSiO_2_ structure was designed and prepared in two processes hydrothermal and temperature. This effectivecatalyst was applied for the synthesis of pyrimidoquinolinesas pure target product in high yields. This catalytic system has a low catalyst loading, short reaction times, solvent‐free conditions and comfortable product separation by external magnet. The catalyst characterizations were carried out by FT‐IR, XRD, Fe‐SEM, EDX, Mapping, VSM, HR‐TEM and BET techniques.

## Experimental Section

### Chemicals and apparatus

All chemicals and reagents, namely calcium nitrate trihydrate (99 %), nickel nitrate hexahydrate (99 %), iron nitrate nonahydrate (99 %), tetraethyl orthosilicate, glucose, barbituric acid, 1‐naphthylamine and the respective benzaldehyde derivatives, were purchased from Merck Chemical Company. ^1^H NMR spectra were taken with 400 MHz NMR spectrometer for solution NMR analysis. IR spectra were obtained with Shimadzu's instrument. Electrothermal Programmable Digital Melting Point Apparatus used to measure melting points. BGMN/Profex/AutoQuan software reported the results of the X‐Ray Diffraction study. nanoparticles were examined using a field emission scanning electron microscope (FE‐SEM), On the Zeiss, that ran at an accelerating voltage of 15 kV. The magnetic properties of nanostructure were measured by a vibrating sample magnetometer (VSM) with PPMS‐9 T at 300 K. The Brunauer‐Emmett‐Teller (BET) specific area and pore volume were measured via the nitrogen adsorption/desorption isotherms method using BELSORP MINI II. High Resolution Transmission Electron Microscopy (HR‐TEM) was prepared by a FEI TECNAI F20 at 200 kV instrument.

### Preparation of the NiCuFe_2_O_4_@mSiO_2_ nanocatalyst

First, glucose (4.95 mg) in 25 mL H_2_O, Fe(NO_3_)_3_ ⋅ 9H_2_O (0.8 mg), Ni(NO_3_)_2_ ⋅ 6H_2_O (0.14 mg), and Cu(NO_3_)_2_ ⋅ 3H_2_O solution (0.12 mg) were dispersed in 10 mL distilled water. After being stirred for about 30 min, the mixture was transferred into a Teflon‐lined stainless steel autoclave. The autoclave was maintained at 180 °C for 24 h and then naturally cooled to ambient temperature. The precipitate was filtered off, washed with water and ethanol, then dried at 100 °C and NiCuFe_2_O_4_@C was obtained. In the second step, NiCuFe_2_O_4_@C (0.1 g), CTAB (0.15 g), and NH_3_ ⋅ H_2_O (580 μL) were dispersed in to the mixture of 40 mL H_2_O and ethanol under magnetic stirring for 30 min. Then, 150 μL TEOS were added to the mixture reaction and the reaction was maintained for 6 h at room temperature. A black solid was obtained, washed with water and ethanol, then dried in the oven at 70 °C and calcined for 3 h at 600 °C. The final, multi yolk@shell NiCuFe_2_O_4_@mSiO_2_ was thus obtained.

### General procedure for the primidoquinolines

In this work, 1‐naphthylamine (1.0 mmol), 4‐Br‐benzaldehyde (1.0 mmol), barbituric acid (1 mmol) and NiCuFe_2_O_4_ (1 mg) are mixed together in the round bottomed flask on the stirred magnetically for 20 min at 50 °C. The progress of the reaction was monitored by thin‐layer chromatography (TLC). After completion of the reaction, EtOH (3 mL) was added and the catalyst was separated by an external magnet. The crude products were obtained by recrystallization in ethanol to give the pure product.

**4′,4′,6,6‐Tetramethyl‐3‐phenyl‐3,5,6,7‐phenyl‐7,12‐dihydro‐11H‐benzo[h]pyrimido[4,5‐b]quinoline‐8,10‐dione** (**4 a**); white solid; m. p.: >300 °C (lit. m. p.: >300 °C);^[31]^ IR (KBr) υ=3335, 1705, 1649, 1545, 1483 cm^−1^; ^1^H NMR (400 MHz, DMSO‐d_6_): δ=5.18 (s, H), 7.08 (t, H, *J*=4.0 Hz), 7.17–7.30 (m, 4H), 7.51 (dd, 2H, *J*_1_=8.0 Hz, *J*_2_=10.0) 7.65 (t, H, *J*=4.0 Hz), 7.86 (d, H, *J*=4.0 Hz), 7.92 (d, H, *J*=4.0 Hz), 9.06 (s, H, NH), 10.1 (s, H, NH), 10.68 (s, H, NH) ppm.

**7‐(2‐Chloro‐phenyl)‐7,12‐dihydro‐11H‐benzo[h]pyrimido[4,5‐b]quinoline‐8,10‐dione** (**4 b**); white solid; m. p.: >300 °C (lit. m. p.: >300 °C);^[31]^ IR (KBr) υ=3396,1707,1644, 1549, 1395, 741 cm^−1^; ^1^H NMR (400 MHz, DMSO‐d_6_): δ=0.85 (s, 3H), 1.16 (s, 9H), 1.59(s, 1H), 1.99 (d, H,*J*=4.0 Hz), 2.17 (d, H, *J*=4.0 Hz), 2.22 (dd, 2H, *J_1_* =4.0 Hz, *J_2_* =10.0 Hz), 2.54–2.58(m, H,), 2.67 (s, H), 3.06(d, H), 4.38(s, H), 7.04(s, H), 7.16(s, H), 7.26(s, 2H) ppm.

**7‐(*p*‐Tolyl‐phenyl)‐7,12‐dihydro‐11H‐benzo[h]pyrimido[4,5‐b]quinoline‐8,10‐dione** (**4 c**); white solid; m. p.: >300 °C (lit. m. p.: >300 °C)^[31]^ IR (KBr) υ=3411, 1642, 1547, 1394, 518 cm^−1^; ^1^H NMR(400 MHz, DMSO‐d _6_): δ=2.17 (s, 3H), 5.13 (s, H), 6.99 (d, 2H, *J*=4.0 Hz), 7.13 (d, H, *J*=4.0 Hz), 7.27 (d, H, *J*=4.0 Hz), 7.51 (dd, 3H, *J_1_* =4.0 Hz, *J_2_* =10.0 Hz), 7.63 (d, H, *J*=2.0 Hz), 7.85 (d, H, *J*=4.0 Hz), 7.92 (H, d, *J*=4.0 Hz), 9.04 (s, H, NH), 9.99 (s, H, NH), 10.66 (s, H, NH) ppm.

**7‐(2‐Fluoro‐phenyl)‐7,12‐dihydro‐11H‐benzo[h]pyrimido[4,5‐b]quinoline‐8,10‐dione** (**4 d**); white solid; m. p.: >300 °C; IR (KBr) υ=3336, 3169, 3071, 1607, 1550, 1482, 754 cm^−1^; ^1^H NMR (DMSO‐d_6_, 400 MHz): δ=5.50 (s, H), 7.04–7.08 (m, 2H), 7.17–7.22 (m, 2H), 7.31 (s, H), 7.48–7.56 (m, 2H), 7.66 (s, H), 7.86 (d, H, *J*=4.0 Hz), 7.92(d, H, *J*=4.0 Hz), 9.10 (s, H, NH), 10.00(s, H, NH), 10.69 (s, H, NH) ppm.

**7‐(4‐Fluoro‐phenyl)‐7,12‐dihydro‐11H‐benzo[h]pyrimido[4,5‐b]quinoline‐8,10‐dione** (**4 e**); cream‐coloured solid; m. p.: >300 °C (lit. m. p.: >300 °C);^[31]^ IR (KBr) υ=3423, 3341, 3070, 1704, 1608, 1549, 749 cm^−1^; ^1^H NMR (400 MHz, DMSO‐d_6_) 5.21 (s, 3H), 7.01 (t, 2H, *J*=4.0 Hz), 7.26–7.29 (m, 2H), 7.49–7.55 (m, 3H), 7.65 (t, H, *J*=4.0 Hz), 7.86 (d, H, *J*=4.0), 7.92 (d, H, *J*=4.0), 9.07 (s, H, NH), 10.00 (s, H, NH), 10.69 (s, H, NH) ppm.

**7‐(4‐Hydroxy‐phenyl)‐7,12‐dihydro‐11H‐benzo[h]pyrimido[4,5‐b]quinoline‐8,10‐dione** (**4 f**); white solid; m. p.: >300 °C (lit. m. p.: >300 °C);^[31]^ IR (KBr) υ=3343, 1650, 1457,1457, 1266, 802 cm^−1^;^1^H NMR (400 MHz, DMSO‐d_6_): 5.55 (s, H), 6.61 (t, H, *J*=4.0 Hz), 6.75 (d, H, *J=*4.0 Hz), 6.87–6.91 (m, 2H), 7.39 (d, H, *J*=4.0 Hz), 7.45–7.53 (m, 2H), 7.63 (t, H, *J*=4.0 Hz), 7.84 (d, H, *J*=4.0), 7.90 (d, H, *J*=4.0 Hz), 9.05 (s, H, NH), 9.67 (s, H, NH), 10.03 (s, H, OH), 10.75 (s, H, NH) ppm.

**7‐(3‐Bromo‐phenyl)‐7,12‐dihydro‐11H‐benzo[h]pyrimido[4,5‐b]quinoline‐8,10‐dione** (**4 g**); white solid; m.p: >300 °C (lit. m. p.: >300 °C);^[31]^ IR (KBr): υ=3337,1708, 1649,1544, 559 cm^−1^; ^1^H NMR (400 MHz, DMSO‐d_6_): 5.22 (s, H), 7.16 (t, H, *J*=4.0 Hz), 7.24 (d, H, *J*=4.0 Hz), 7.30 (d, H, *J*=4.0 Hz), 7.47–7.56 (m, 3H), 7.66 (t, H, *J*=4.0 Hz), 7.87 (d, H, *J*=4.0 Hz), 7.93 (d, H, *J*=4.0 Hz), 9.09 (s, H, NH), 10.03 (s, H, NH), 10.73(s, H, NH) ppm.

**7‐(4‐Chloro‐phenyl)‐7,12‐dihydro‐11H‐benzo[h]pyrimido[4,5‐b]quinoline‐8,10‐dione** (**4 h**); white solid; m. p.: >300 °C (lit. m. p.: >300 °C);^[31]^ IR (KBr) υ=3419, 1710, 1609, 1484, 568 cm^−1^; ^1^H NMR (400 MHz, DMSO‐d_6_): 5.21(s, 3H), 7.24–7.29 (m, 5H), 7.52 (dd, *J*=8.0 Hz, *J*=10.0 Hz), 7.65 (t, H, *J*=4.0 Hz), 7.87 (d, H, *J*=4.0 Hz), 7.92 (d, H, *J*=4.0 Hz), 9.08(s, H, NH), 10.01(s, H, NH), 10.68 (s, H, NH) ppm.

**7‐(3‐Nitro‐phenyl)‐7,12‐dihydro‐11H‐benzo[h]pyrimido[4,5‐b]quinoline‐8,10‐dione** (**4 i**); orange solid; m. p.: >300 °C;IR (KBr) υ=3246, 1712, 1550, 1478, 1346, 806 cm^−1^; ^1^H NMR (400 MHz, DMSO‐d_6_): 5.43 (s, H), 7.32(d, H, *J*=6.0 Hz), 7.50–7.58 (m, 3H), 7.65–7.73 (m, 2H), 7.87 (d, H, *J*=4.0 Hz), 7.94 (t, 2H, *J*=4.0 Hz), 8.13 (s, H), 9.16 (s, H, NH), 10.06 (s, H, NH), 1.075 (s, H, NH) ppm.

**7‐(4‐Nitro‐phenyl)‐7,12‐dihydro‐11H‐benzo[h]pyrimido[4,5‐b]quinoline‐8,10‐dione** (**4 j**);orange solid;m. p.: >300 °C (lit. m. p.: >300 °C);^[31]^ IR (KBr) υ=3372, 1711, 1650, 1516, 1344, 755 cm^−1^; ^1^H NMR (400 MHz, DMSO‐d_6_) 5.39 (s, H), 7.27 (d, H, *J*=4.0 Hz), 7.49–7.57 (m, 3H), 7.67 (d, 2H, *J*=4.0 Hz), 7.87 (d, H, *J*=4.0 Hz), 7.93 (d, H, *J*=6.0 Hz), 8.09 (d, H, *J*=4.0 Hz), 9.16 (s, H, NH), 10.05 (s, H, NH), 10.75 (s, H, NH) ppm.

**7‐(4‐Bromo‐phenyl)‐7,12‐dihydro‐11H‐benzo[h]pyrimido[4,5‐b]quinoline‐8,10‐dione** (**4 k**); white solid; m. p.: >300 °C; IR (KBr) υ=3382, 3249, 2971, 1710, 1599, 1544, 675 cm^−1^; ^1^H NMR (400 MHz, DMSO‐d_6_) 5.20 (s, H), 7.22 (d, H, *J*=4.0 Hz), 7.26 (d, H, *J*=4.0 Hz), 7.39 (d, 2H, *J*=4.0 Hz), 7.48–7.54 (m, 3H), 7.64(q, H, *J*=8.0 Hz), 7.86 (d, H, *J*=4.0 Hz), 7.92 (d, H, *J*=4.0 Hz), 9.08 (s, H, NH), 10.00 (s, H, NH), 10.27 (s, H, NH) ppm.

**7‐(5‐Bromo‐2‐hydroxy‐phenyl)‐7,12‐dihydro‐11H‐benzo[h]pyrimido[4,5‐b]quinoline‐8,10‐dione** (**4 l**); orange solid; m. p.: >300 °C; IR (KBr) υ=3335, 1700, 1643, 1597, 1468, 757 cm^−1^; ^1^H NMR (400 MHz, DMSO‐d_6_) 5.55 (s, H), 6.59–6.74 (d, 2H, *J*=4.0 Hz), 7.02–7.09 (m, H), 7.07 (m, 2H), 7.39–7.66 (m, 4H), 7.85–8.10(m, 2H), 9.07 (s, H, NH), 10.2(s, H, NH), 10.21(s, H, NH), 10.77(s, H, OH) ppm.

**7‐(2,3‐Dihydroxy‐phenyl)‐7,12‐dihydro‐11H‐benzo[h]pyrimido[4,5‐b]quinoline‐8,10‐dione** (**4 m**); white solid, m. p.: >300 °C; IR (KBr) υ=3340, 1717, 1649, 1546, 743 cm^−1^; ^1^H NMR (400 MHz, DMSO‐d_6_) 5.55 (s, H), 6.21 (d, H, *J*=4.0 Hz), 6.42(t, H, *J*=4.0 Hz), 6.50 (d, H, *J*=4.0 Hz), 7.32 (d, H, *J*=4.0 Hz), 7.47–7.54 (m, 2H), 7.63 (t, H, *J*=4.0 Hz), 7.82 (d, H, *J*=6.0 Hz), 7.93 (d, H, *J*=6.0 Hz), 8.98 (s, H, NH), 9.10 (s, 2H, NH), 10.13 (s, H, OH), 10.85 (s, H, OH) ppm.

## Conflict of interest

The authors declare no conflict of interest.

1

## Supporting information

As a service to our authors and readers, this journal provides supporting information supplied by the authors. Such materials are peer reviewed and may be re‐organized for online delivery, but are not copy‐edited or typeset. Technical support issues arising from supporting information (other than missing files) should be addressed to the authors.

Supporting Information

## Data Availability

The data that support the findings of this study are available in the supplementary material of this article.
