# Supplementary material for: Design, Fabrication and Characterization of Multi‐Yolk@Shell NiCuFe2O4@mSiO2 Mesoporous Nanocomposite Spheres for the Synthesis of Pyrimido‐Quinolines under Solvent‐Free Conditions
Source: ChemistryOpen. 2023 Sep 8;12(9):e202300053. doi: 10.1002/open.202300053 (PMC10491931; doi:10.1002/open.202300053)
Supplement: Supplementary file 1 — Supporting Information [file OPEN-12-e202300053-s001.pdf]

ChemistryOpen

Supporting Information

Design, Fabrication and Characterization of Multi-Yolk@Shell $NiCuFe_2O_4$@$mSiO_2$ Mesoporous Nanocomposite Spheres for the Synthesis of Pyrimido-Quinolines under Solvent-Free Conditions

Somayeh Kazempour and Hossein Naeimi*

General multicomponent procedure for the synthesis of pyrimidoquinolines

Pyrimidoquinolines are synthesized from the multi-component reaction. In this work, 1-naphthylamine (1.0 mmol), 4-Br benzaldehyde (1.0 mmol) barbituric acid (1 mmol) and $NiCuFe_2O_4$ (1 mg) are mixed together in the round- bottomed flask on the stirred magnetically for 20 min at 50 C. The progress of the reaction was monitored by thin-layer chromatography (TLC). After completion of the reaction, EtOH (2 ml) was added and the catalyst was separated by an external magnetic. The crude products were obtained by recrystallization in ethanol to give the pure product.

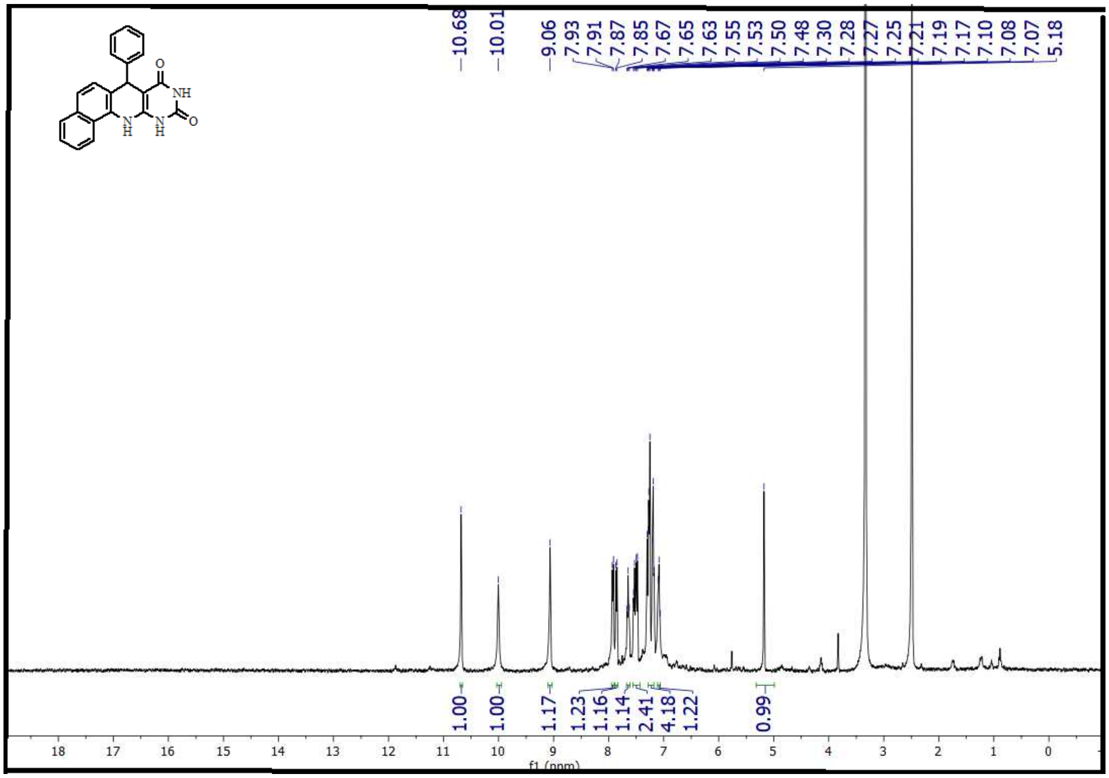

^{1}H NMR spectra of **4a**

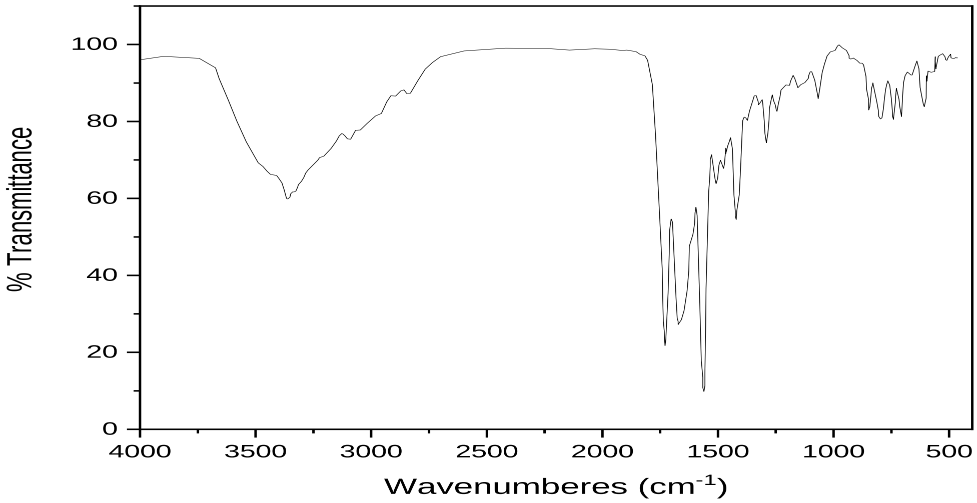

FT-IR of **4a**

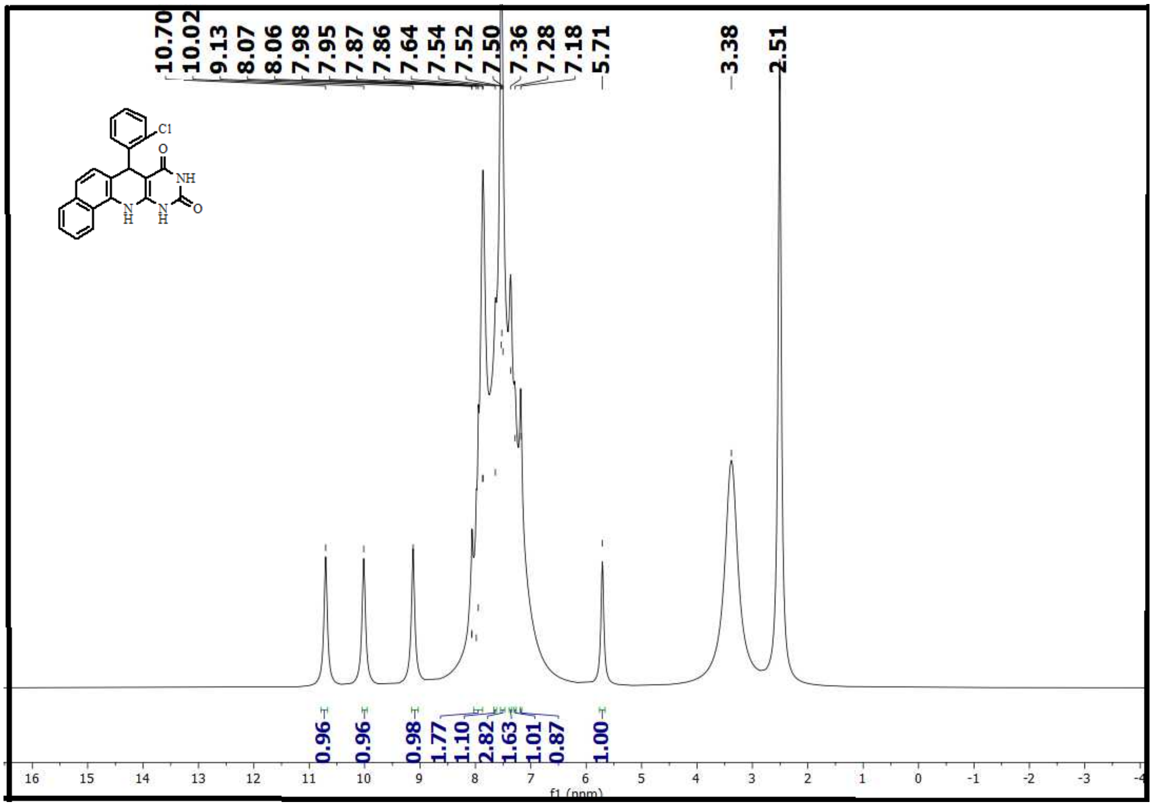

^{1}H NMR spectra of **4b**

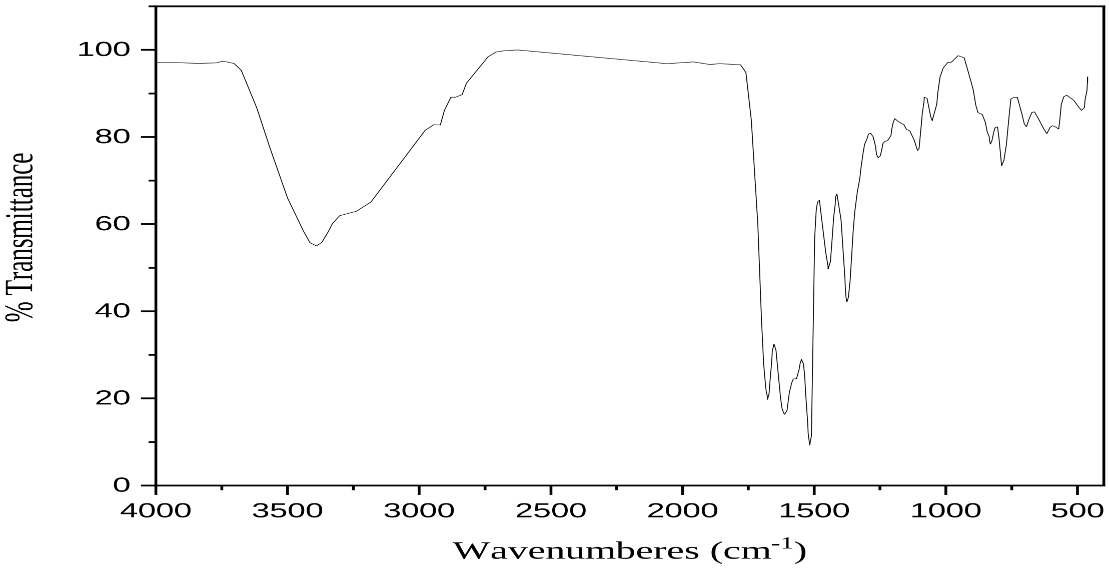

FT-IR of **4b**

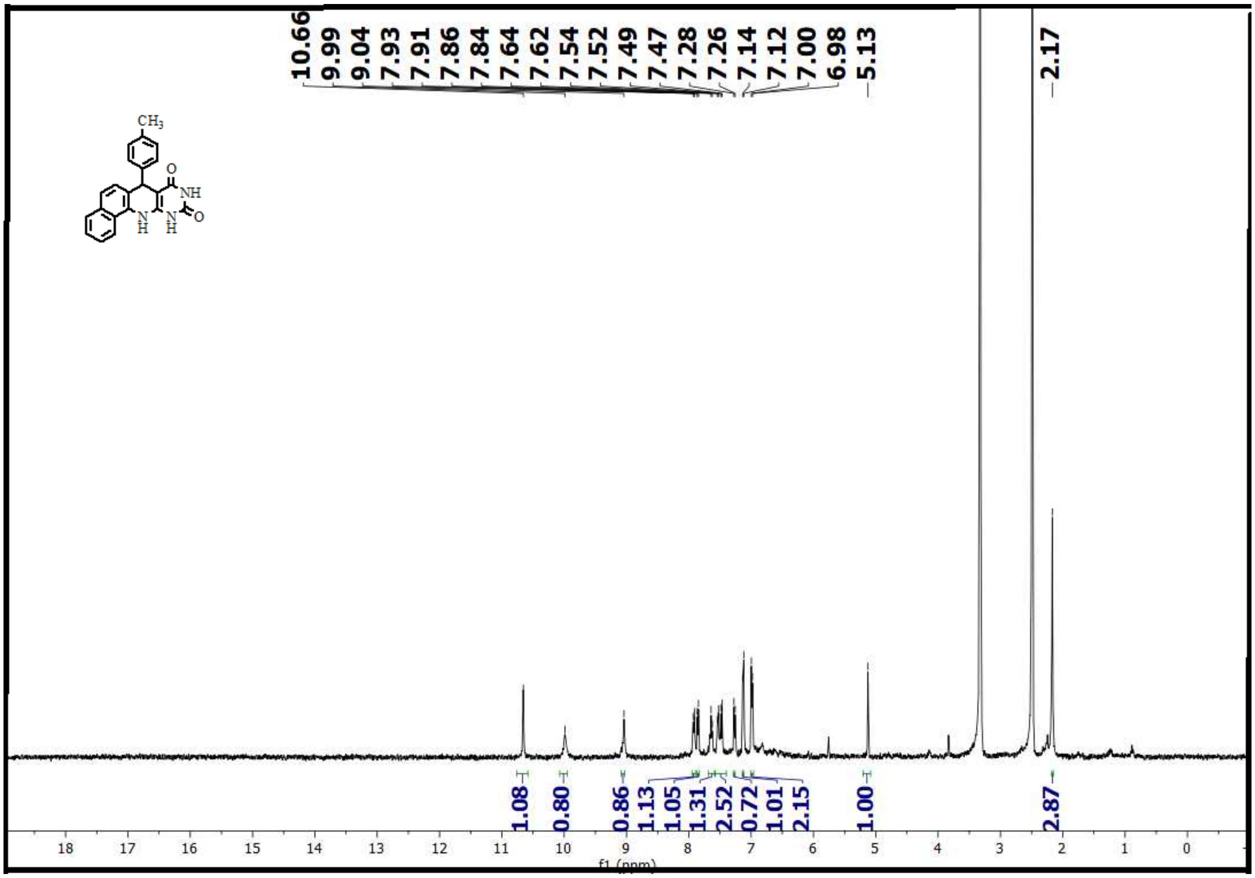

^{1}H NMR spectra of **4c**

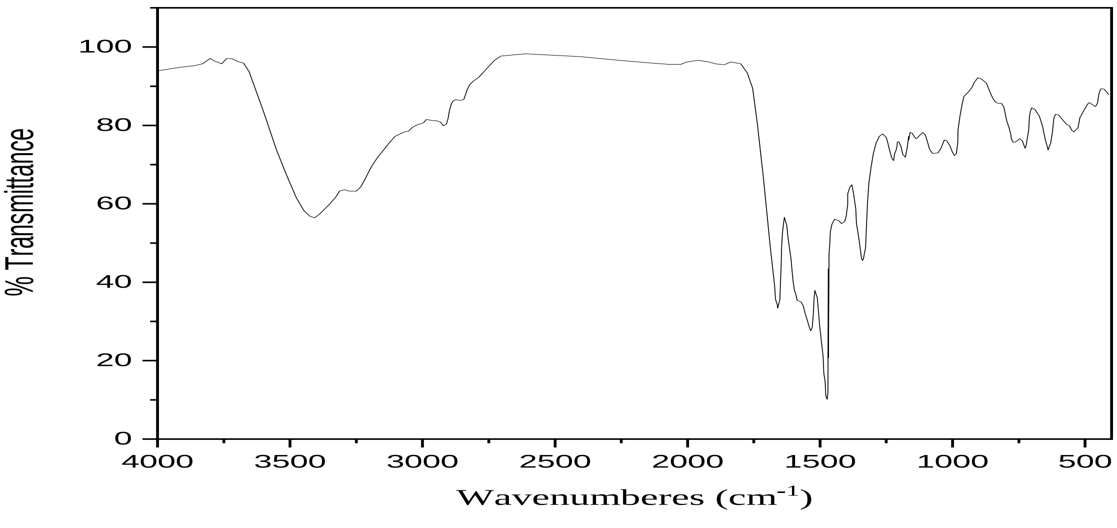

FT-IR of **4c**

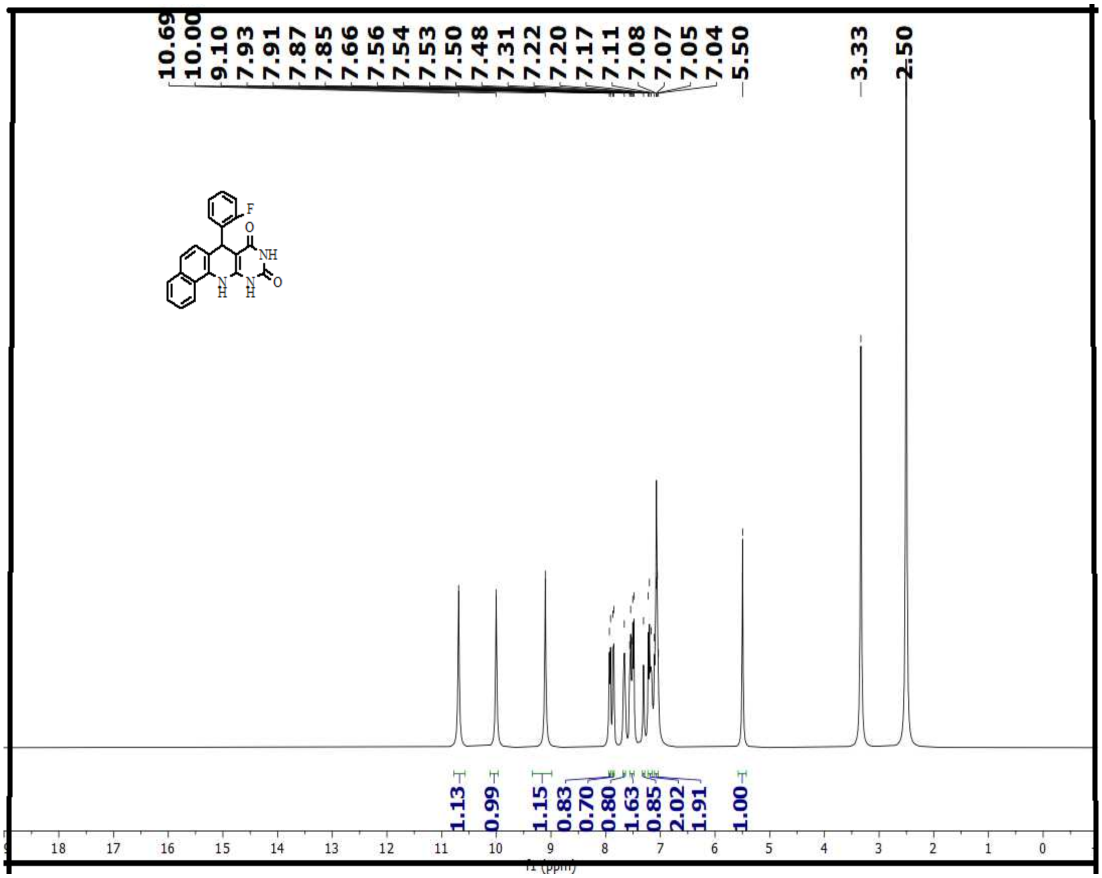

^{1}H NMR spectra of **4d**

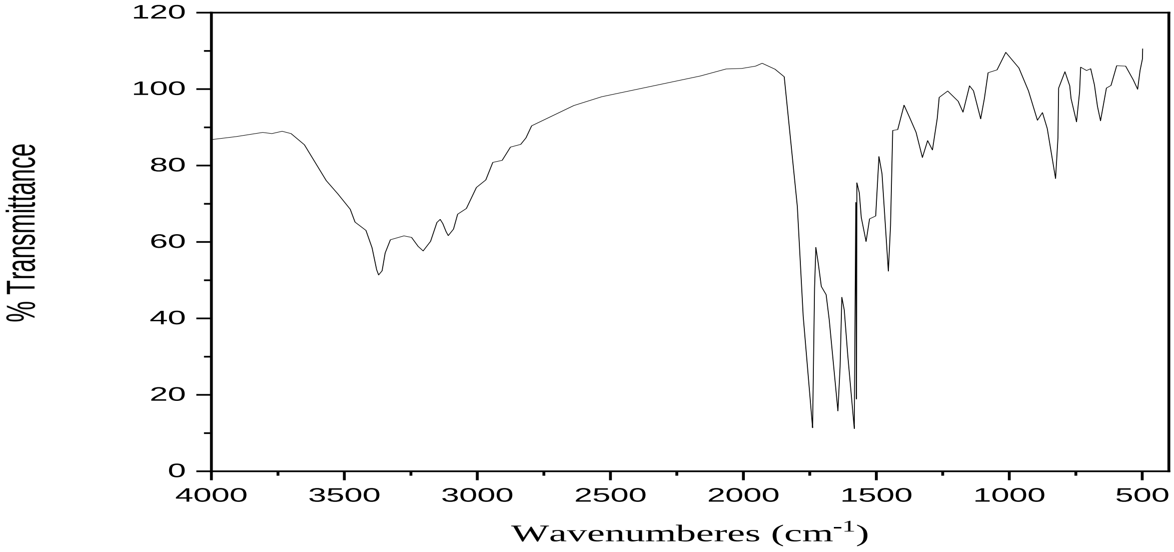

FT-IR of **4d**

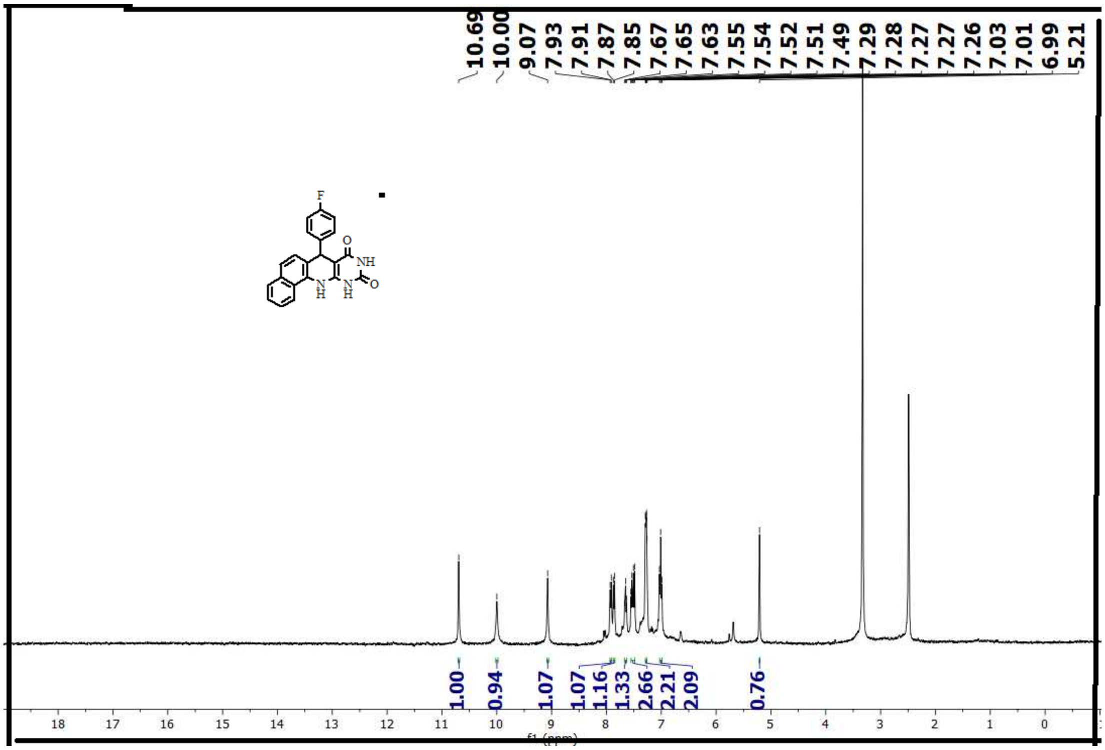

H NMR spectra of **4e**

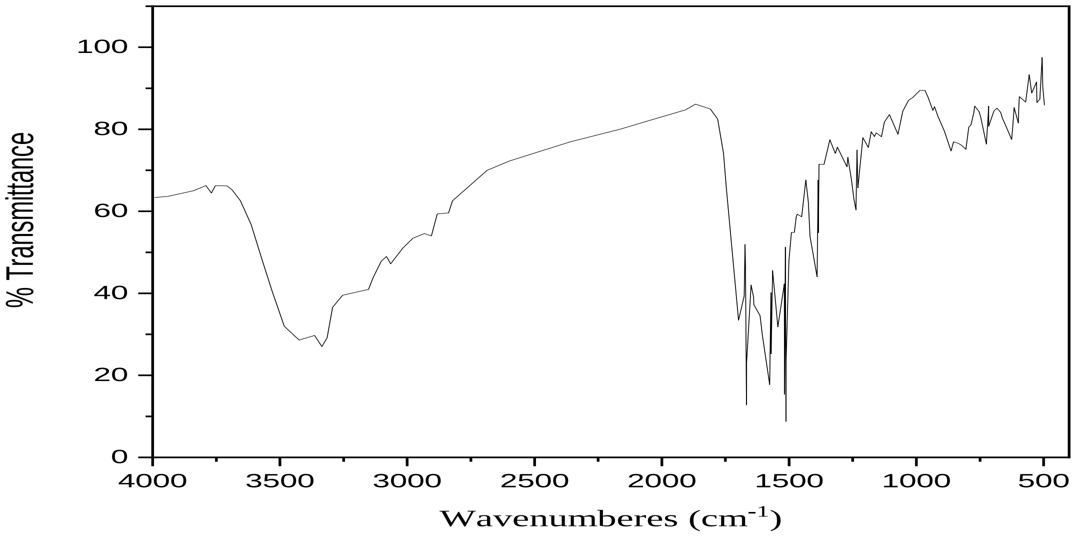

FT-IR of **4e**

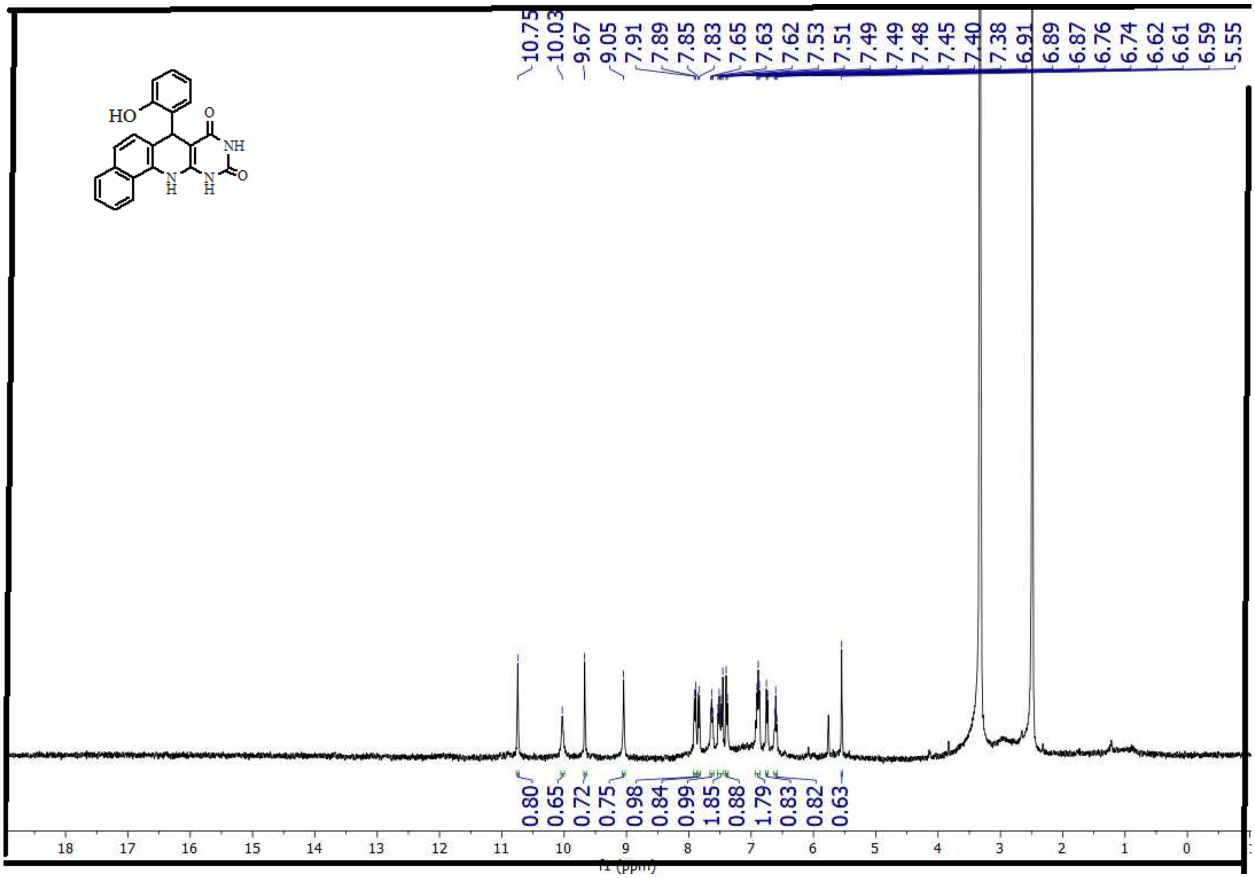

^{1}H NMR spectra of **4f**

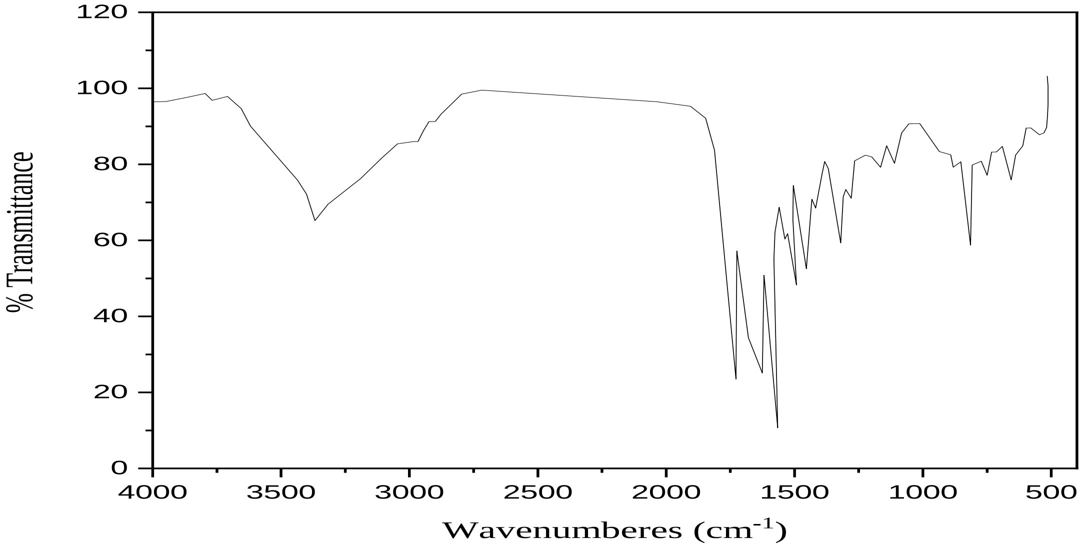

FT-IR of **4f**

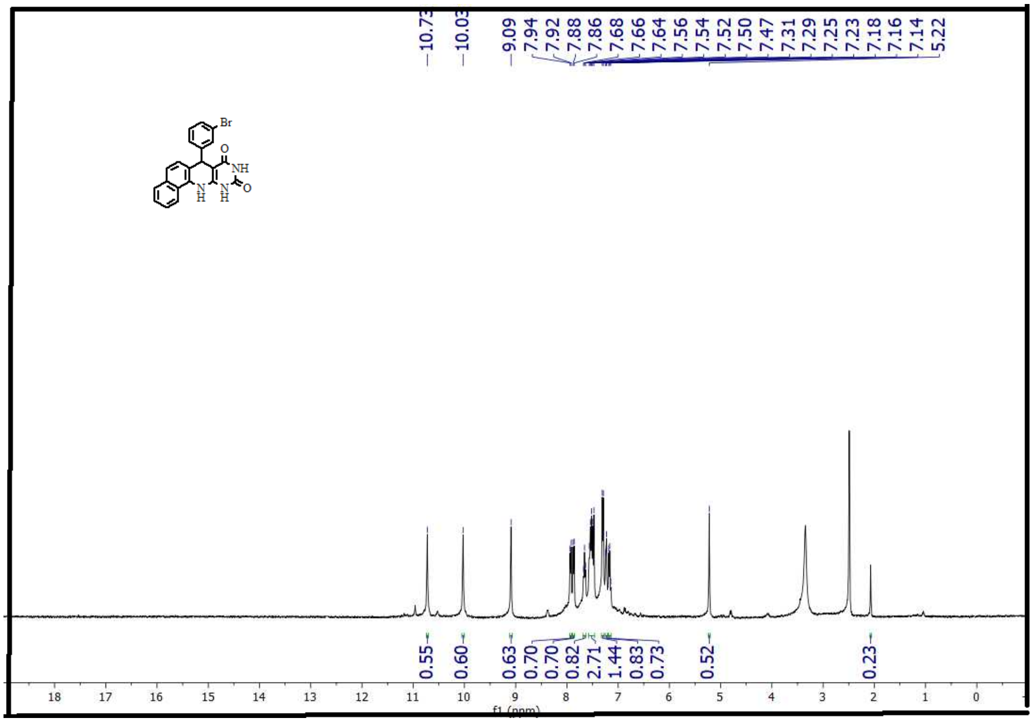

^{1}H NMR spectra of **4g**

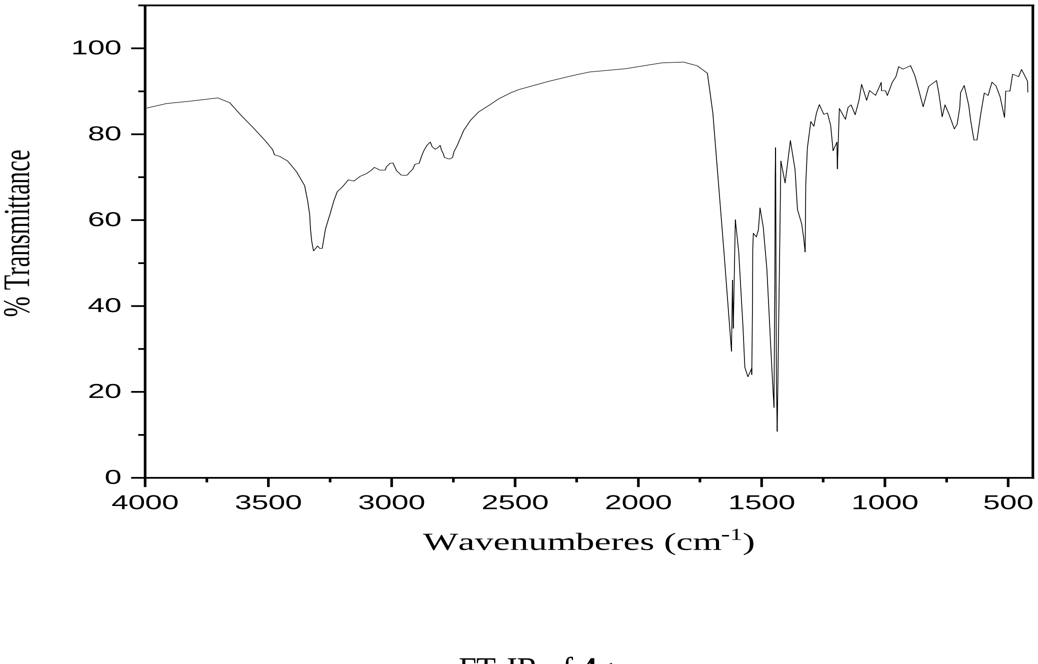

FT-IR of **4g**

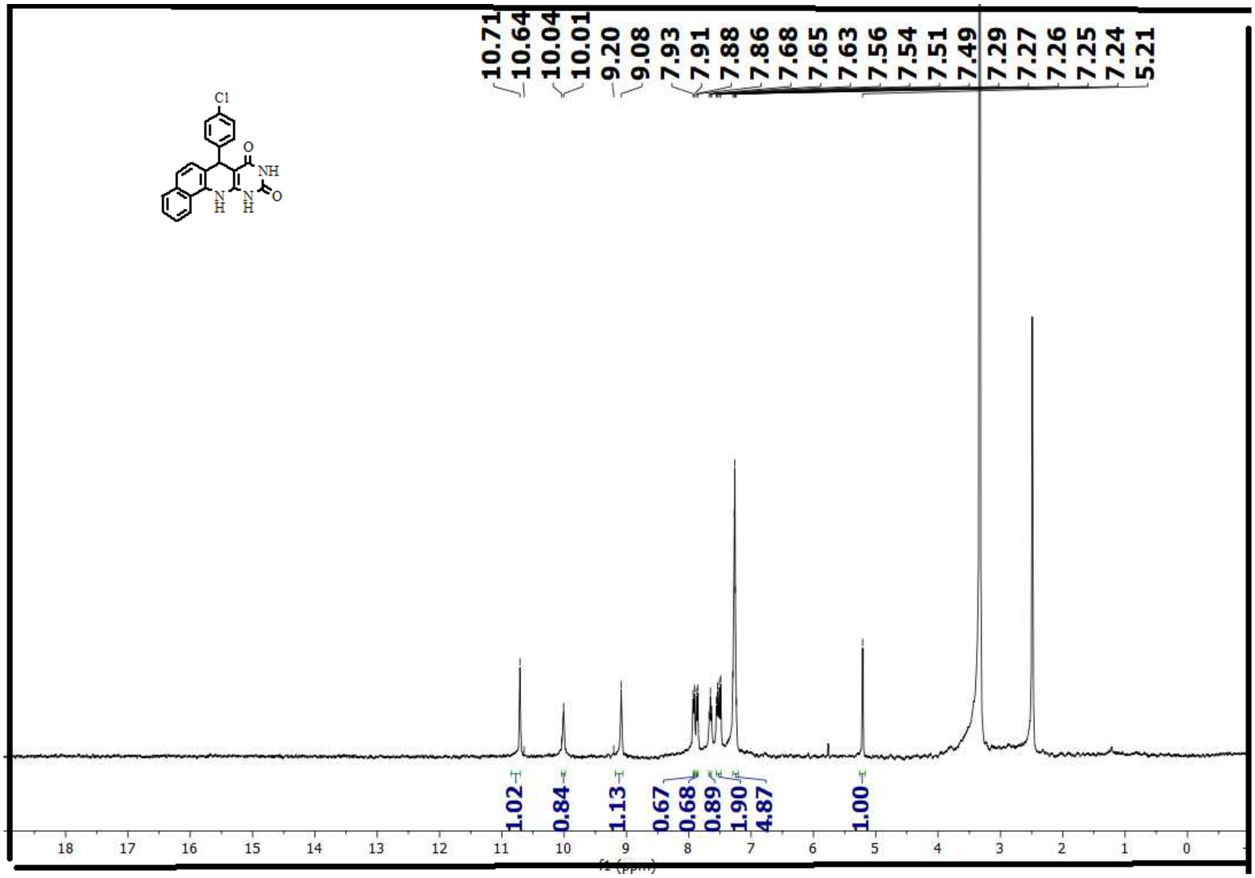

^{1}H NMR spectra of **4h**

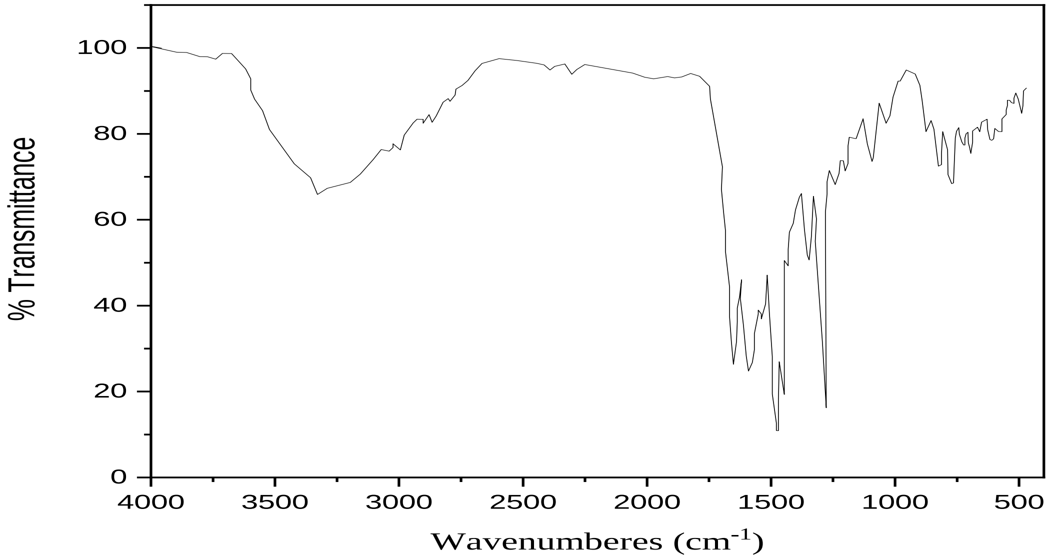

FT-IR of **4h**

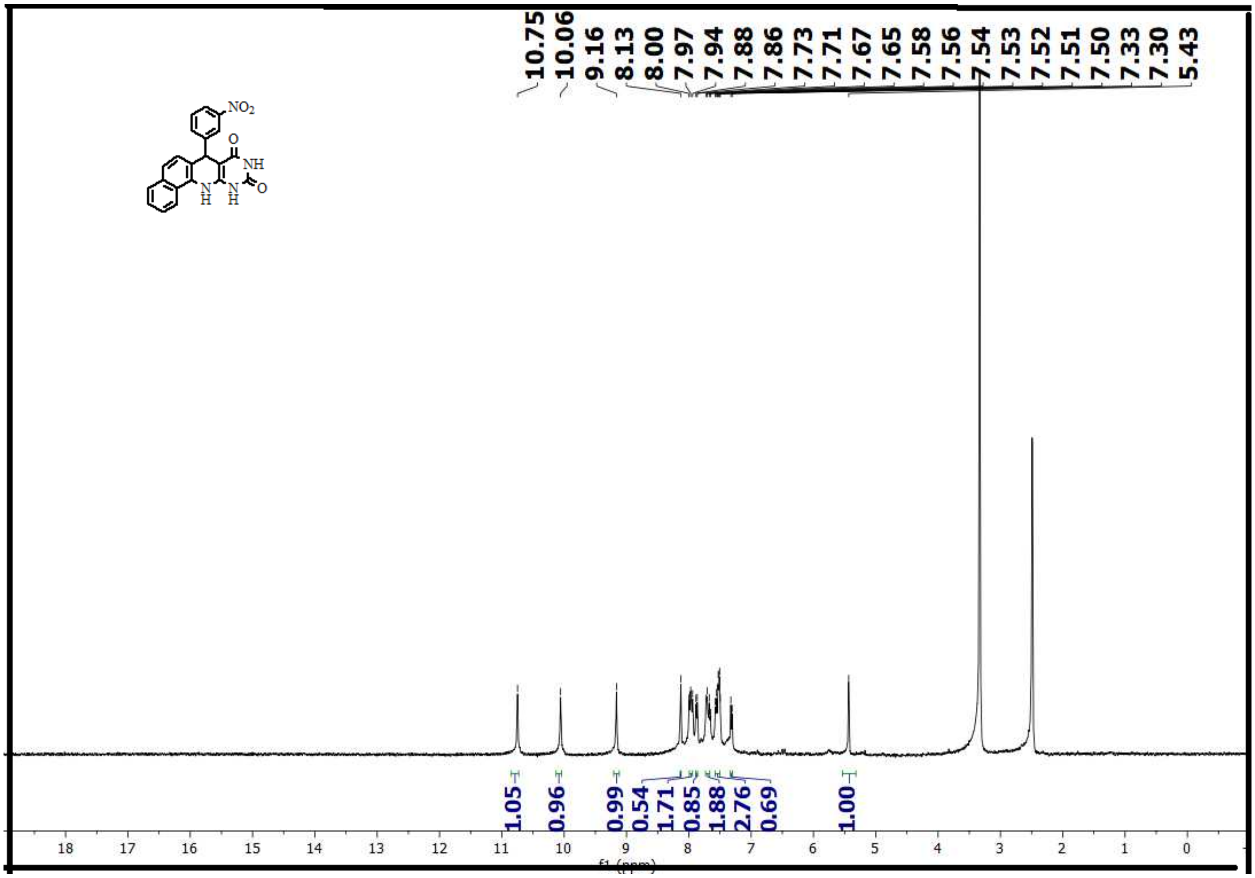

H NMR spectra of **4i**

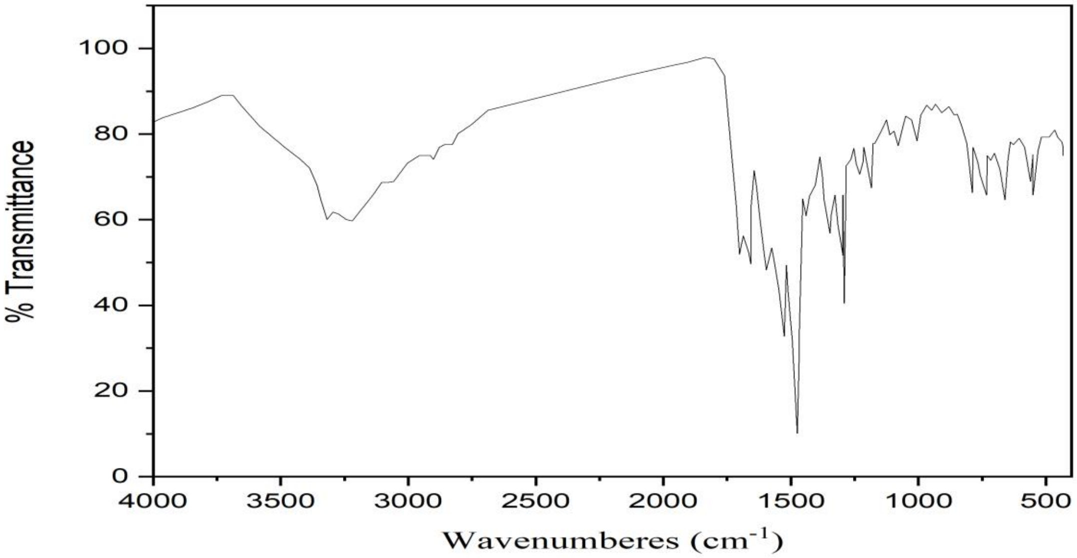

FT-IR of **4i**

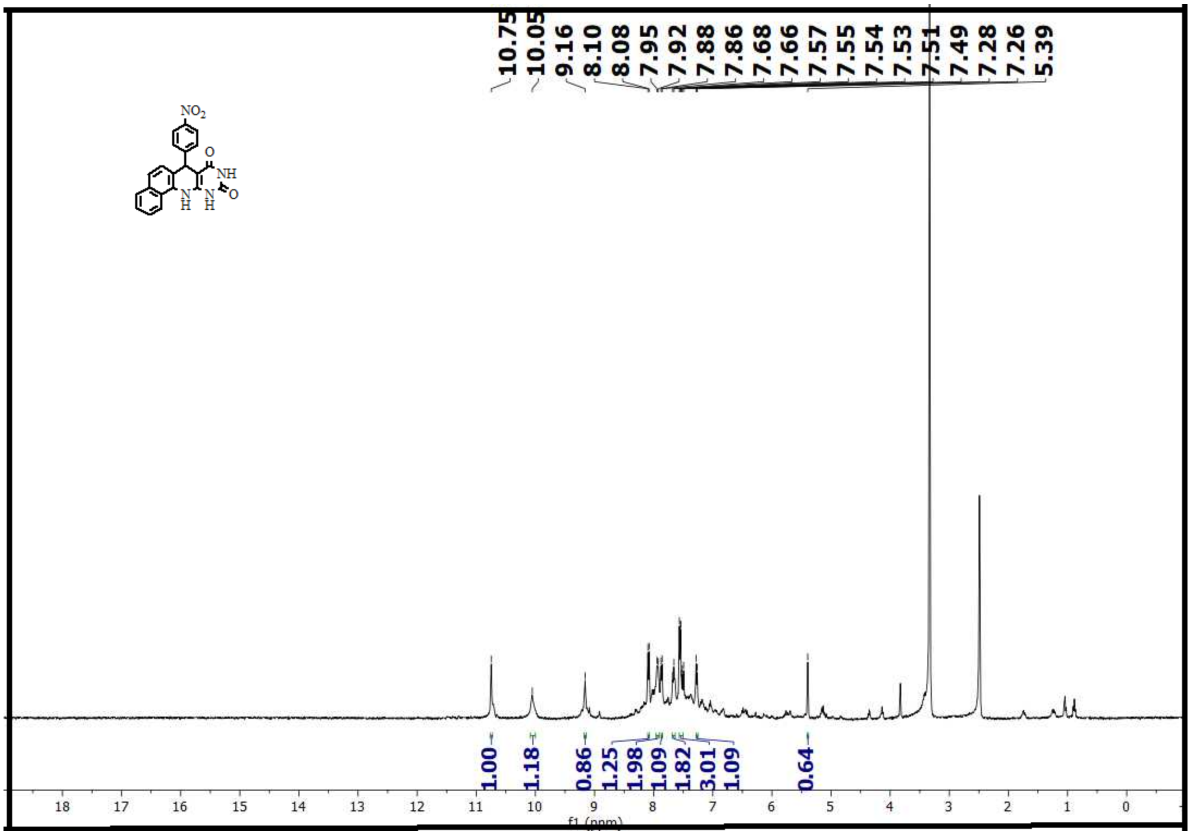

H NMR spectra of **4j**

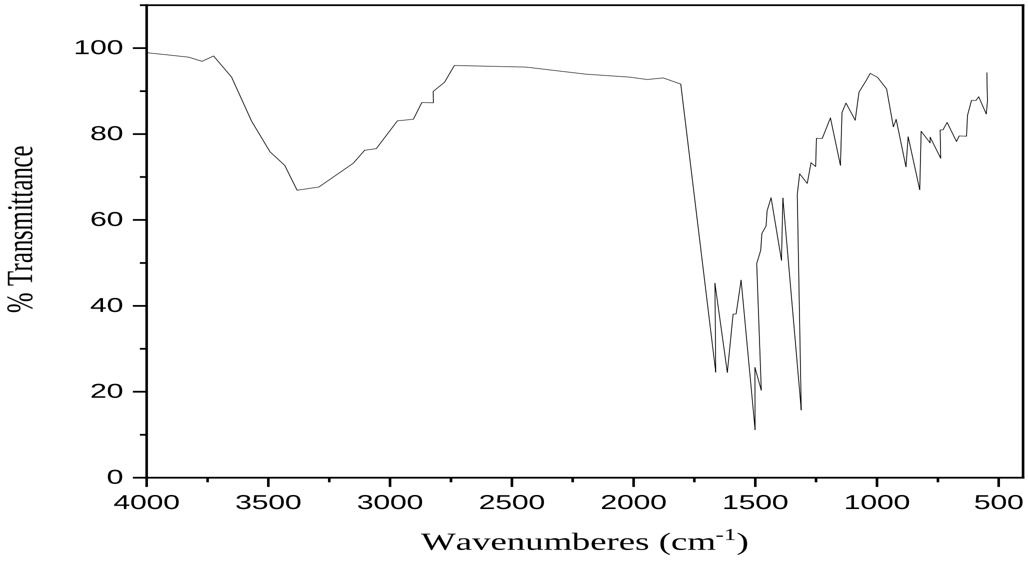

FT-IR of **4j**

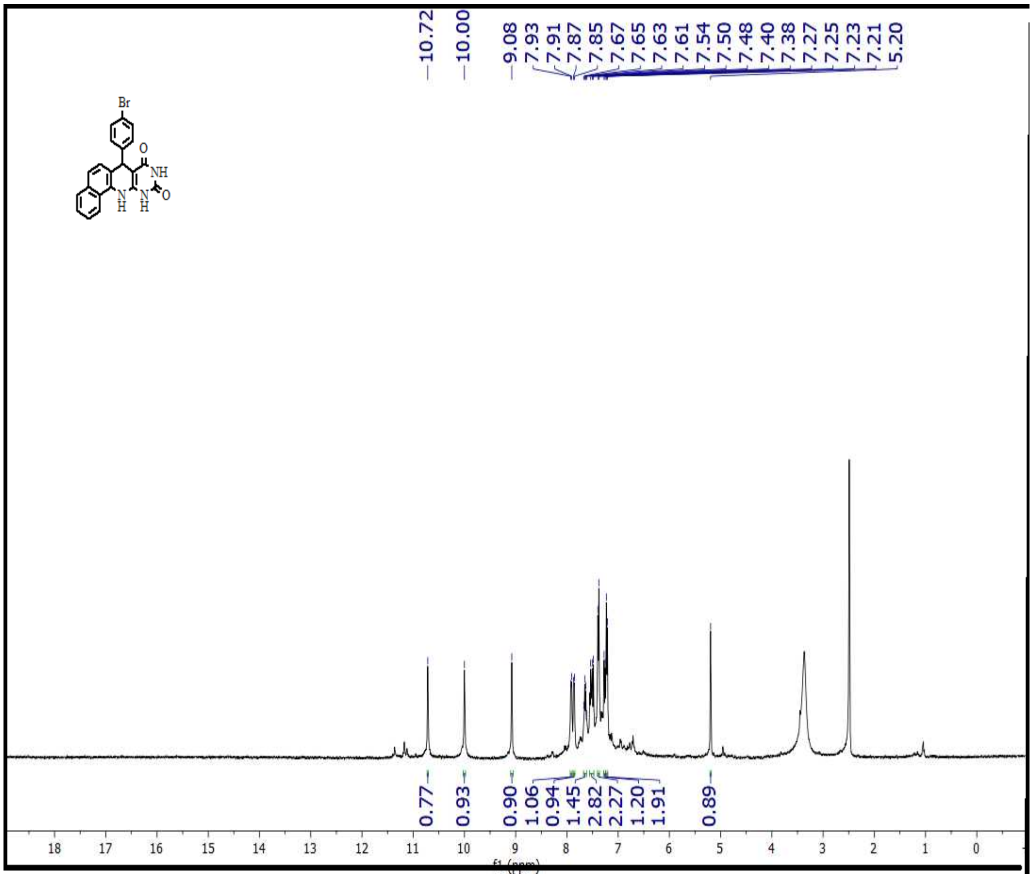

^{1}H NMR spectra of **4k**

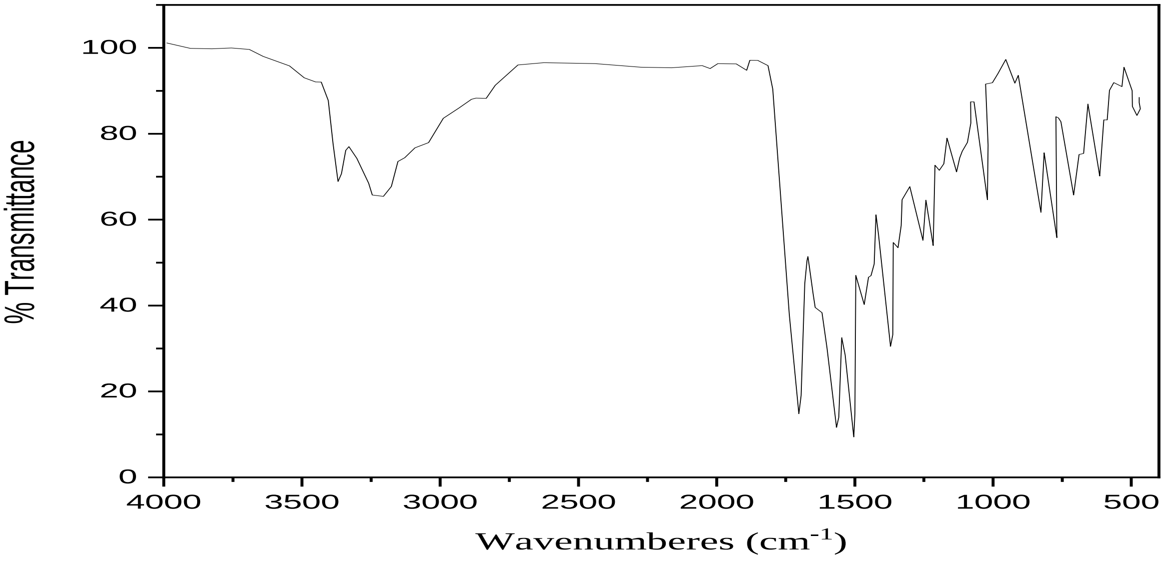

FT-IR of **4k**

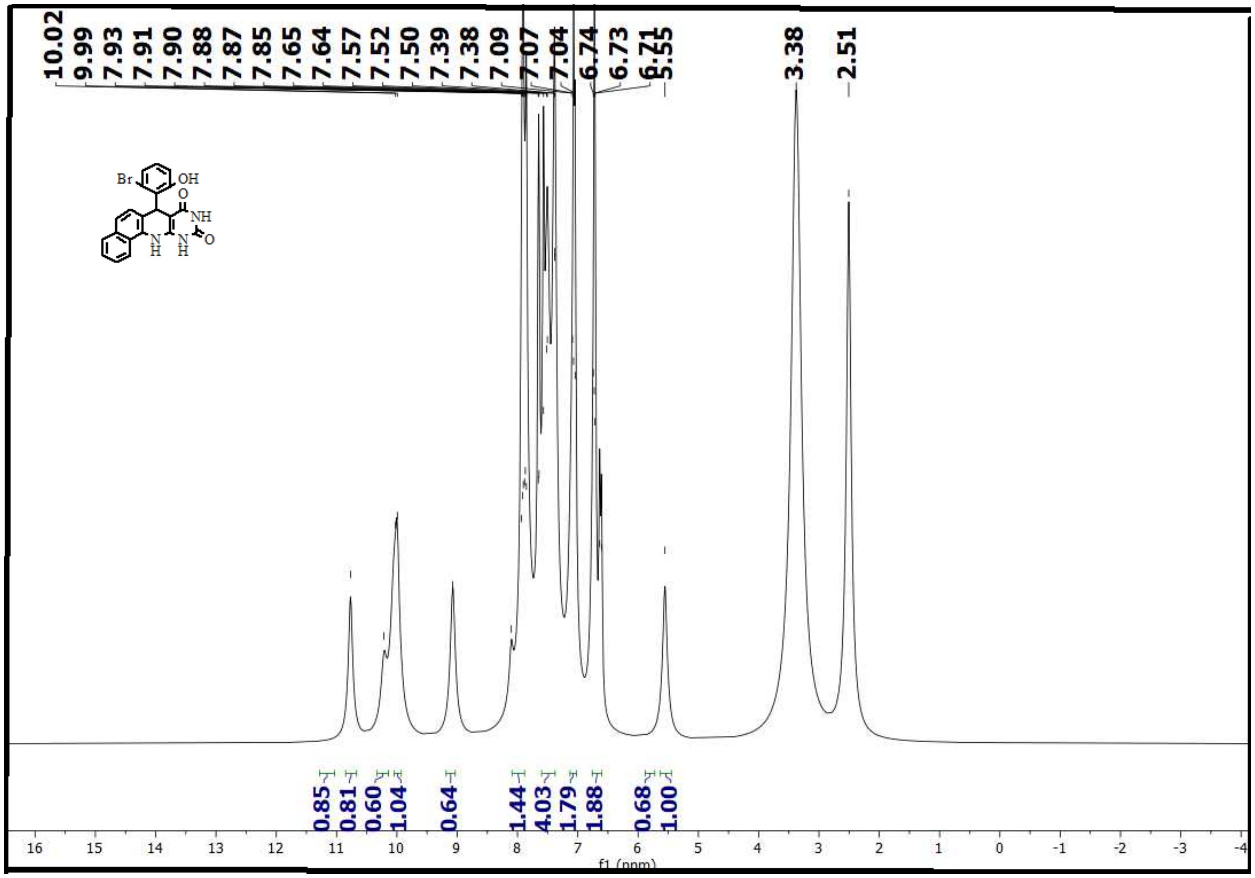

H NMR spectra of **4l**

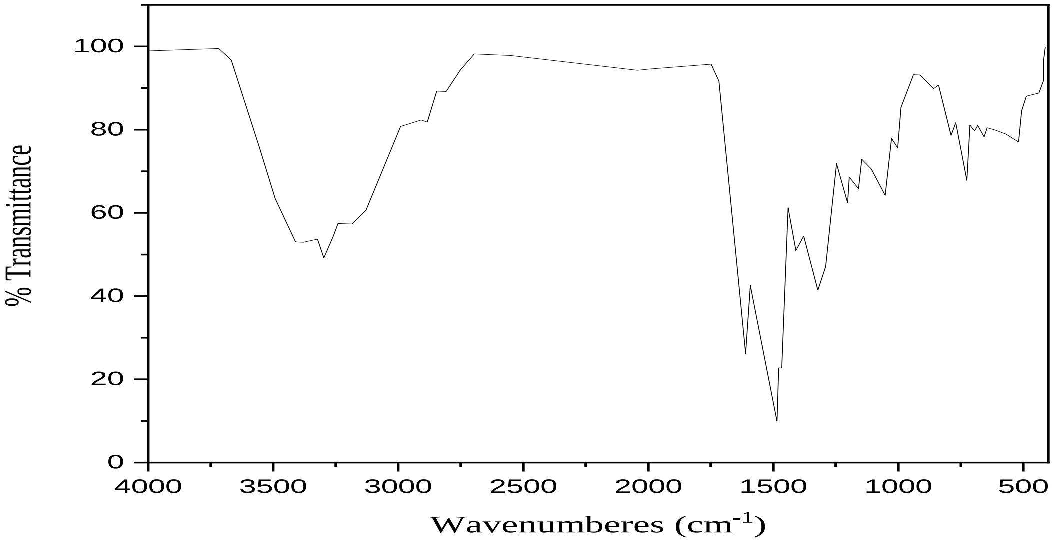

FT-IR of **4l**

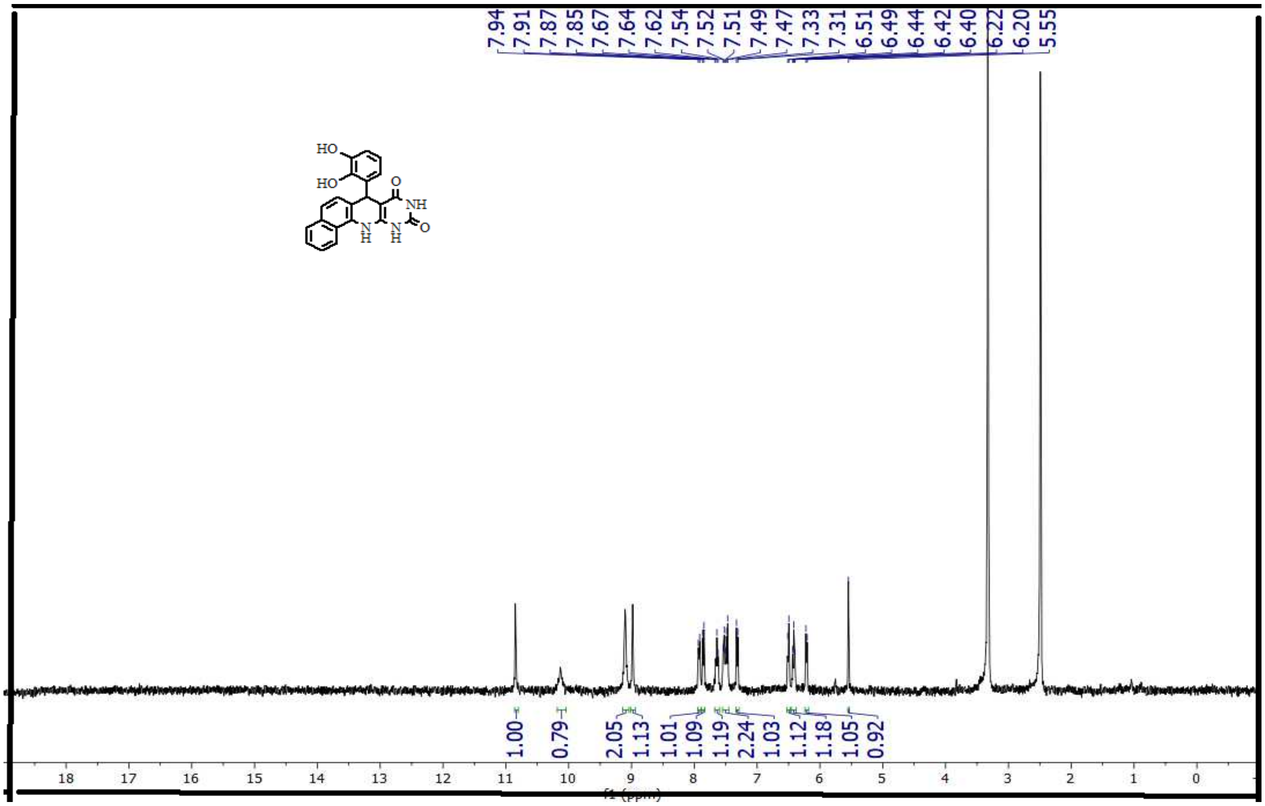

^{1}H NMR spectra of **4m**

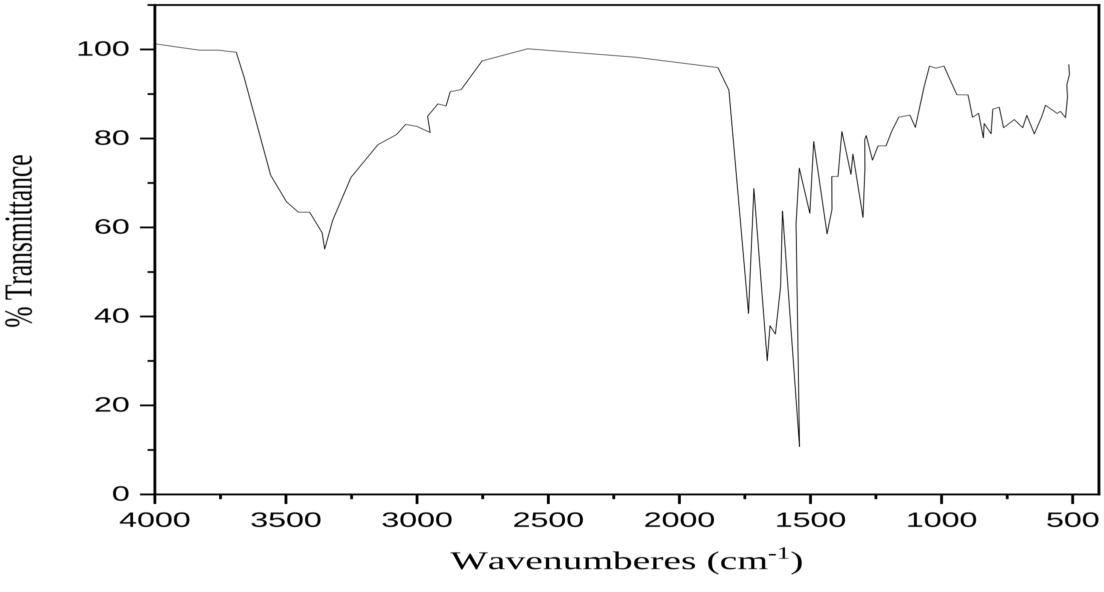

FT-IR of **4m**
